# Isolation and functional analysis of *CONSTANS-LIKE* genes suggests that a central role for CONSTANS in flowering time control is not evolutionarily conserved in *Medicago truncatula*

**DOI:** 10.3389/fpls.2014.00486

**Published:** 2014-09-18

**Authors:** Albert C. S. Wong, Valérie F. G. Hecht, Kelsey Picard, Payal Diwadkar, Rebecca E. Laurie, Jiangqi Wen, Kirankumar Mysore, Richard C. Macknight, James L. Weller

**Affiliations:** ^1^School of Biological Sciences, University of TasmaniaHobart, TAS, Australia; ^2^Department of Biochemistry, University of OtagoDunedin, New Zealand; ^3^Plant Biology Division, Samuel Roberts Noble FoundationArdmore, OK, USA

**Keywords:** legume, flowering, photoperiod, Medicago, CONSTANS

## Abstract

The zinc finger transcription factor *CONSTANS* has a well-established central role in the mechanism for photoperiod sensing in Arabidopsis, integrating light and circadian clock signals to upregulate the florigen gene *FT* under long-day but not short-day conditions. Although *CONSTANS*-LIKE (*COL*) genes in other species have also been shown to regulate flowering time, it is not clear how widely this central role in photoperiod sensing is conserved. Legumes are a major plant group and various legume species show significant natural variation for photoperiod responsive flowering. Orthologs of several Arabidopsis genes have been shown to participate in photoperiodic flowering in legumes, but the possible function of *COL* genes as integrators of the photoperiod response has not yet been examined in detail. Here we characterize the *COL* family in the temperate long-day legume *Medicago truncatula*, using expression analyses, reverse genetics, transient activation assays and Arabidopsis transformation. Our results provide several lines of evidence suggesting that *COL* genes are unlikely to have a central role in the photoperiod response mechanism in this species.

## Introduction

The length of the daily photoperiod is an important environmental variable that influences plant development. The most widely-recognized response to photoperiod is the induction of flowering, but photoperiod also controls other vegetative and reproductive characteristics, including formation of storage organs, axillary branching, and vegetative bud dormancy (Thomas and Vince-Prue, 1997). Within individual species, genetic variation for photoperiod responsiveness can be a major feature of adaptation to different latitudes and is therefore significant both in the natural environment and for agriculture.

As a result, there is widespread interest in the mechanism by which plants measure and respond to photoperiod, and this has been extensively examined in both Arabidopsis and rice. The study of induced mutants and natural variants affecting photoperiod responsiveness in both species has identified genes in the *FT* florigen family as the major target of photoperiod regulation, and have highlighted the general importance of light signaling pathways and the circadian clock for photoperiod measurement (Andres and Coupland, 2012; Brambilla and Fornara, 2013; Song et al., 2013; Tsuji et al., 2013).

In Arabidopsis, one gene in particular, *CONSTANS (CO)*, has a central role in the mechanism of photoperiod measurement, integrating clock and light signals to provide photoperiod-specific induction of FT expression (Andres and Coupland, 2012; Song et al., 2013). *CO* was originally defined on the basis of a long day (LD)-specific late-flowering mutant phenotype (Koornneef et al., 1991), and encodes a B-box zinc finger transcription factor (Putterill et al., 1995). Transgenic plants overexpressing *CO* are extremely early flowering, and epistatic and regulatory interactions position *CO* genetically between *GI* and *FT* (Onouchi et al., 2000; Suárez-López et al., 2001). It has subsequently been shown that *FT* is an early transcriptional target of *CO* (Samach et al., 2000), and that the CO protein binds to the *FT* promoter (Tiwari et al., 2010).

The LD-specificity for activation of *FT* by *CO* is achieved through regulation of CO protein abundance at both transcriptional and post-translational level. *CO* mRNA is rhythmically expressed under the control of the circadian clock, such that peak expression occurs at night under short days (SD) but in the afternoon under LD (Suárez-López et al., 2001). Afternoon *CO* expression in LD is reinforced by action of the FKF1 blue light photoreceptor, which interacts with GI to degrade CDF proteins, which are transcriptional repressors of CO (Fornara et al., 2009; Song et al., 2012). CO protein accumulation is prevented in darkness by the ubiquitin ligase COP1 (Jang et al., 2008) but permitted in the afternoon under LD where phyA suppresses COP1 activity (Valverde et al., 2004) and FKF1 directly stabilizes CO (Song et al., 2012).

In rice, a warm-season crop with a short-day requirement for flowering, the *CO*-like gene *Hd1* also contributes to photoperiod measurement and photoperiod-specific regulation of *FT* family genes (Brambilla and Fornara, 2013). In contrast to Arabidopsis *CO*, *Hd1* appears to be a bifunctional regulator, acting to promote *FT* expression in SD and to repress it in LD (Izawa et al., 2002; Kojima et al., 2002). These observations have suggested that *CO* function may be widely conserved across the angiosperms. This conclusion has been tested in expression and functional analyses in a number of other species. In some species such as potato and sugar beet, *CO*-like genes do seem to be involved in photoperiod responses (Chia et al., 2008; Gonzalez-Schain et al., 2012), whereas evidence from other species such as barley, and poplar is less clear or inconclusive (Campoli et al., 2012; Hsu et al., 2012).

In the legume species pea (*Pisum sativum* L.), cloning of several flowering loci has demonstrated conserved roles for Arabidopsis circadian clock genes *GI*, *ELF4* and *ELF3* in the regulation of *FT* genes and the control of photoperiod-responsive flowering (Hecht et al., 2007; Liew et al., 2009; Weller et al., 2012). A similar role has also been demonstrated for *GI* in soybean (Watanabe et al., 2011). However, the endogenous function of *CO*-like (*COL*) genes in legumes has not been directly tested, and the possibility that they may participate in photoperiod measurement is still unresolved. In this study we have examined the potential involvement of *COL* genes in photoperiodic flowering of the temperate long-day legume *Medicago truncatula*, using expression analyses, Arabidopsis complementation, and loss-of-function mutants.

## Materials and methods

### Plant material

The experiments shown in **Figures 2, 4** used the *Medicago truncatula* line R108 and derived mutants obtained from reverse-screening the *Tnt1* insertion population described by Tadege et al. (2008). The Medicago sequences used for the experiments in **Figure 3** were obtained from cv Jester (*MtFTa1* promoter, *MtCOLa-d*) or R108 (*MtCOLe-h*).

### Growth conditions

Arabidopsis plants were grown under long day photoperiod (16 h light/8 h dark) in growth cabinets maintained at 21°C with 30% to 40% humidity, and an irradiance of approximately 115 μmol m^−2^ s^−1^. Medicago plants were grown in growth cabinets maintained at 22°C under either long (16-h) or short-day (8-h) photoperiods.

### Expression analysis

Analysis of *MtCOL* expression followed procedures described by Hecht et al. (2011). Harvested material consisted of all expanded leaves from three-week-old plants, with each sample consisting of material pooled from two plants. Two technical replicates and three biological replicates were performed for each timepoint. Transcript levels for experimental genes were evaluated as previously described (Weller et al., 2009), relative to the reference gene *MtTEF1*α. Primer sequences are given in Supplemental Table 2.

### Arabidopsis transformation

DNA fragments containing full-length coding sequences of *MtCOLa-COLh* were amplified by PCR from cDNA and cloned into the pCR8/GW/TOPO TA vector (Invitrogen). The resulting entry vector was then recombined into plant transformation vector, pB2GW7 (Karimi et al., 2002) to generate the *35S:MtCOLa-h* constructs. Transgenic plants were produced by applying *Agrobacterium tumefaciens* strain LBA4404 containing the pB2GW7 vectors to *Arabidopsis co-2* mutant flowers using the protocol described by Martinez-Trujillo et al. (2004). Seeds from these plants were collected and sown directly onto soil and selected using Basta herbicide. Putative transformants were confirmed by qRT-PCR analysis.

### Transient assays

The transient expression assays were performed by infiltrating *Nicotiana benthamiana* leaves, as described by Hellens et al. (2005). Agrobacterium strains containing either the FT promoter–reporter construct or a *35S:COL* construct were co-infiltrated into leaves using a mixture of the two strains at a ratio of 7:1, respectively. Firefly luciferase and Renilla luciferase were assayed 4 d after infiltration using the Dual-Luciferase Reporter Assay System (Promega) as described by Hellens et al. (2005).

## Results

### Defining the *CONSTANS-LIKE (COL)* gene family in legumes

**Figure 1:**
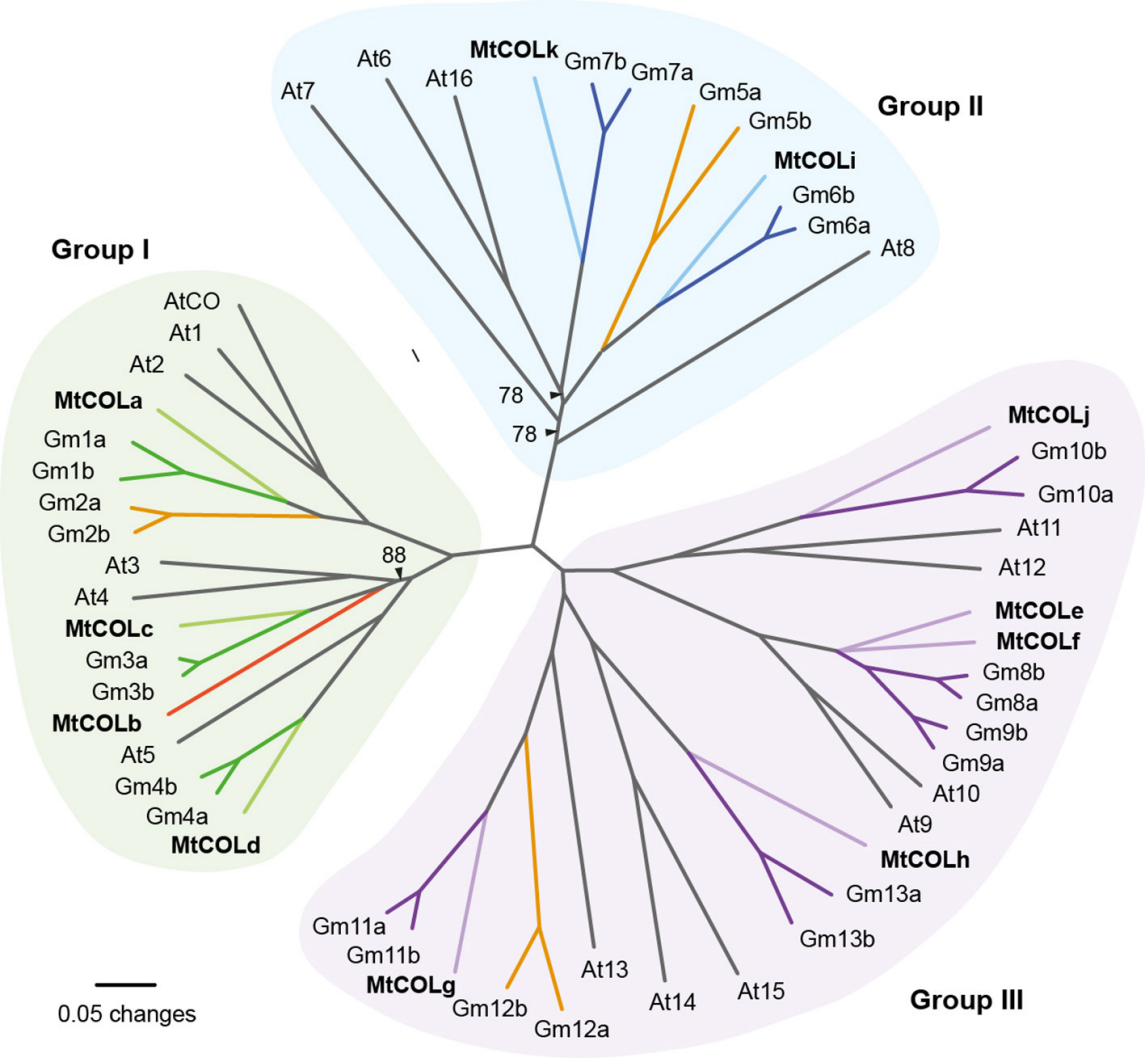
**The *CONSTANS-LIKE* (*COL*) gene family in Medicago**. Phylogram of legume and Arabidopsis COL protein sequences. The analysis is based on the sequence alignment shown in Supplemental Figure 1 online. Sequence details are available in Supplemental Table 1 online. Groups I (green shading), II (blue shading) and III (purple shading) correspond to the classification of Griffiths et al. (2003). Branches representing legume proteins are shaded in color consistent with each group, with soybean proteins shown in dark and Medicago genes in light shading. Branches shown in orange indicate soybean homeolog pairs for which no corresponding Medicago ortholog was found, and the single Medicago gene without a soybean counterpart is shown in red. Branches with bootstrap values <50% have been collapsed, and black arrowheads indicate branches with support >50% but <90%. All other branches have support >90%. At, *Arabidopsis thaliana*; Gm, *Glycine max*; Mt, *Medicago truncatula*.

We previously reported a partial characterization of the *COL* gene family in legumes (Hecht et al., 2005) focusing on the so-called Group I *COL* genes (Griffiths et al., 2003). This group of genes includes Arabidopsis *CO* and is characterized by two B-box domains within an N-terminal Zn finger region, and a conserved C-terminal (CCT) domain that is also found in the circadian clock-related pseudo-response regulator gene *TOC1* and related *PRR* (Strayer et al., 2000; Griffiths et al., 2003). To extend our understanding of legume *COL* genes, we used a combination of database searches and PCR-based approaches to isolate additional *COL* genes in *Medicago truncatula.* We identified a total of 11 expressed and apparently full-length *COL* coding sequences (Figure 1, Supplemental Figure 1) that included four Group I genes (*COLa-COLd*), two group II genes (*COLi, COLk*) and four Group III genes (*COLe-COLh, COLj*). It thus appears that all major groups within the *COL* family are represented in legumes, but some degree of independent expansion has occurred within Groups II and III.

Consistent with a previous report (Hecht et al., 2005) we identified only a single group Ia gene in Medicago (*MtCOLa*) and found that the three Arabidopsis group Ia genes *AtCO*, *AtCOL1* and *AtCOL2* were more similar to each other than to *MtCOLa*.

A recent report from soybean has identified 26 *COL* genes, representing 13 pairs of homeologs (Wu et al., 2014). For nine of these pairs, we identified a single Medicago ortholog (Figure 1), and the clade containing *GmCOL8a/b* and *COL9a/b* also included two Medicago genes; *MtCOLe* and *COLf*. The *MtCOLb* gene had no corresponding pair of genes in soybean, and three soybean homeolog pairs were not represented by Medicago genes. This latter situation could imply the existence of additional Medicago *COL* genes not represented in the current genome build (Mt4.0), and we were particularly interested in a comparison of the Group Ia genes as this clade contains most of the genes known to have *CO*-like function in other species. In soybean, there are four Group Ia *COL* genes; *GmCOL1a/b* and *COL2a/b*. The single Group Ia gene *MtCOLa* is clearly orthologous to the *GmCOL1a*/*b* pair, implying that Medicago might possess a second Group Ia *COL* gene orthologous to *GmCOL2a/b*. To address the possibility, we examined the genomic regions containing *GmCOL2a* and *COL2b* for evidence of microsynteny with the Medicago genome. Supplemental Figure 2 shows that genes in the *GmCOL2a*/*COL2b* regions showed highest similarity to genes on Medicago chromosome 6, with clear evidence of microsynteny, but there was no *MtCOL* gene in this location, suggesting that this gene may have been lost from the Medicago lineage. Similarly, microsynteny between regions containing *GmCOL5a/b* and another part of Medicago chromosome 6, and between regions containing *GmCOL12a/b* and Medicago chromosome 2 (Supplemental Figure 2) also suggests that orthologs of these genes are also absent from the Medicago genome. We therefore tentatively conclude that the 11 Medicago *COL* genes we have identified represent the entire gene family.

### Diurnal rhythms of *COL* gene expression

In Arabidopsis, the characteristic diurnal mRNA expression rhythm of *CO* is linked to its function in photoperiod measurement. Under SD, *CO* expression peaks in the night and is low throughout the day. Under LD, CO expression increases during the afternoon, and this increase is reinforced by an additional relief from repression through the action of the blue-light photoreceptor FKF1 (Imaizumi et al., 2003). We reasoned that if transcriptional regulation of *COL* genes was similarly important for photoperiod responses in temperate legumes, one or more *COL* genes might show distinctly different expression rhythms in long and short days. We therefore examined the diurnal expression rhythms for eight of the 11 *MtCOL* genes (*COLa*-*COLh*).

**Figure 2:**
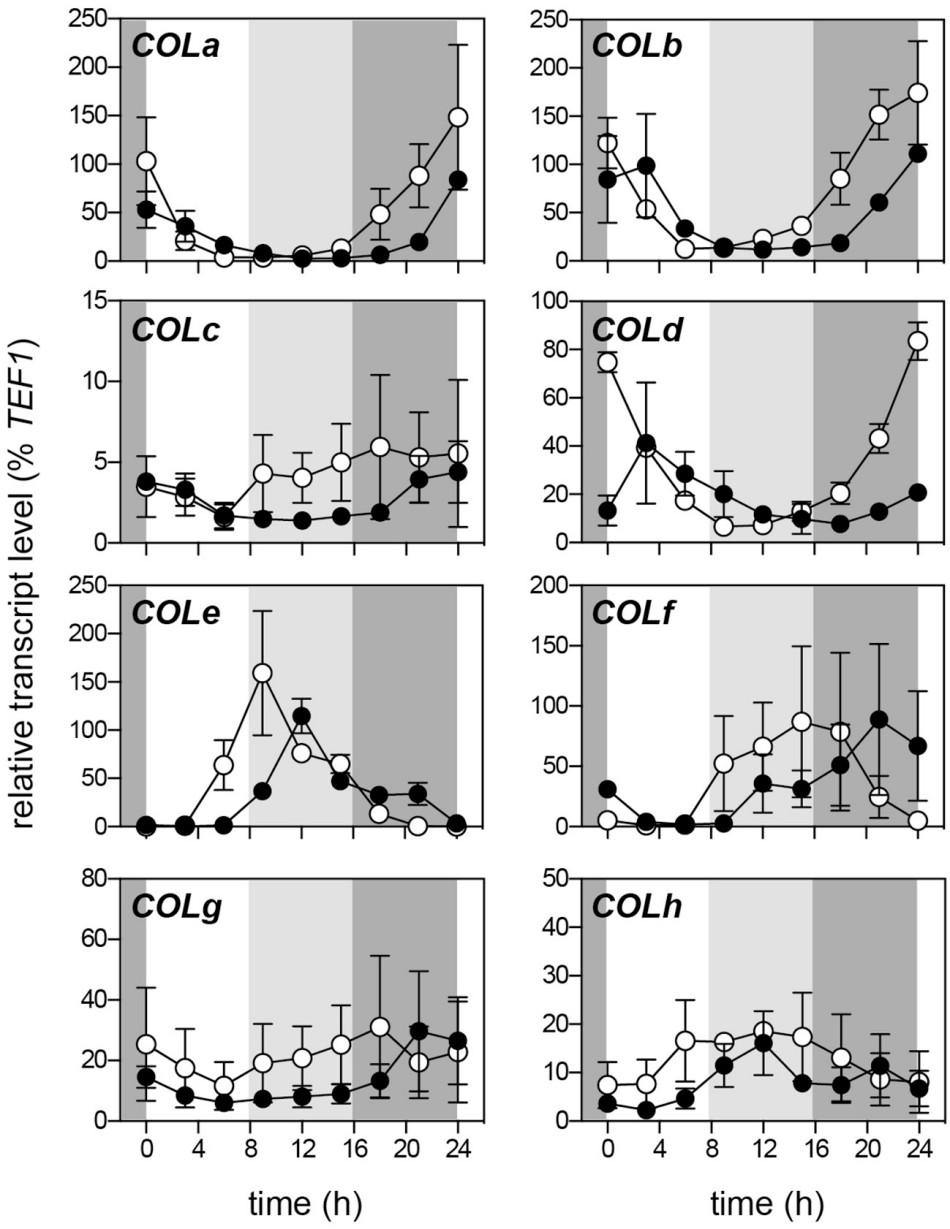
**Rhythmic regulation of *MtCOL* expression under SD and LD**. Transcript levels were determined in fully-expanded leaves taken from 3-week-old R108 seedlings grown under 8-h (short-day; open symbols) or 16-h long-day photoperiods (filled symbols) in growth cabinets at 22°C. The night period common to both treatments is represented by dark gray shading, with the period in which plants are in the light in long days but not short days is represented by light gray shading. Data represent mean ± SE for *n* = 2 biological replicates.

We previously reported that the single group Ia *COL* gene in pea, *PsCOLa*, shows a morning-phased expression rhythm in LD (Hecht et al., 2007) that is similar to the Arabidopsis Group Ia genes *COL1* and *COL2* (Ledger et al., 2001). Figure 2 shows that *MtCOLa* expression also follows a similar LD rhythm with a peak at dawn. The level of expression under SD was not significantly different than under LD throughout the daily timecourse, and under both conditions, *COLa* showed significant morning expression, which declined to basal level by ZT9. Under LD specifically, *COLa* expression remained very low during the afternoon, with no evidence of the afternoon “shoulder” to the LD rhythm that is characteristic of Arabidopsis *CO* (Imaizumi et al., 2003). More generally, there was no evidence for any difference in *COLa* expression during the light phase in LD compared to SD.

Like *COLa*, the Group Ic genes *COLb* and *COLd* also showed a morning-phased rhythm. For both genes, the phase of the expression rhythm was earlier in SD than in LD, typical of the response of many rhythmically-regulated genes to photoperiod. However, as for *COLa*, there was no evidence of a qualitative difference in expression during the light phase between LD and SD conditions for either gene. *COLc* was only expressed at a very low level and showed minimal diurnal variation. In contrast to the Group I genes, the Group III genes generally showed an evening-phased rhythm under LD, which in most cases, was shifted earlier in SD. *COLe* showed the most strongly rhythmic expression with an afternoon peak in LD at around ZT12, and *COLf* expression was also clearly rhythmic, with peak expression under LD during the night. *COLg* and *COLh* were at most weakly rhythmic. The closest similarity to the Arabidopsis *CO* rhythm was seen for *COLf*, which was not expressed at dawn or at either of the two timepoints during the light phase under SD, but showed significant expression at dawn and during the afternoon in LD.

### Activity of legume *COL* genes

**Figure 3:**
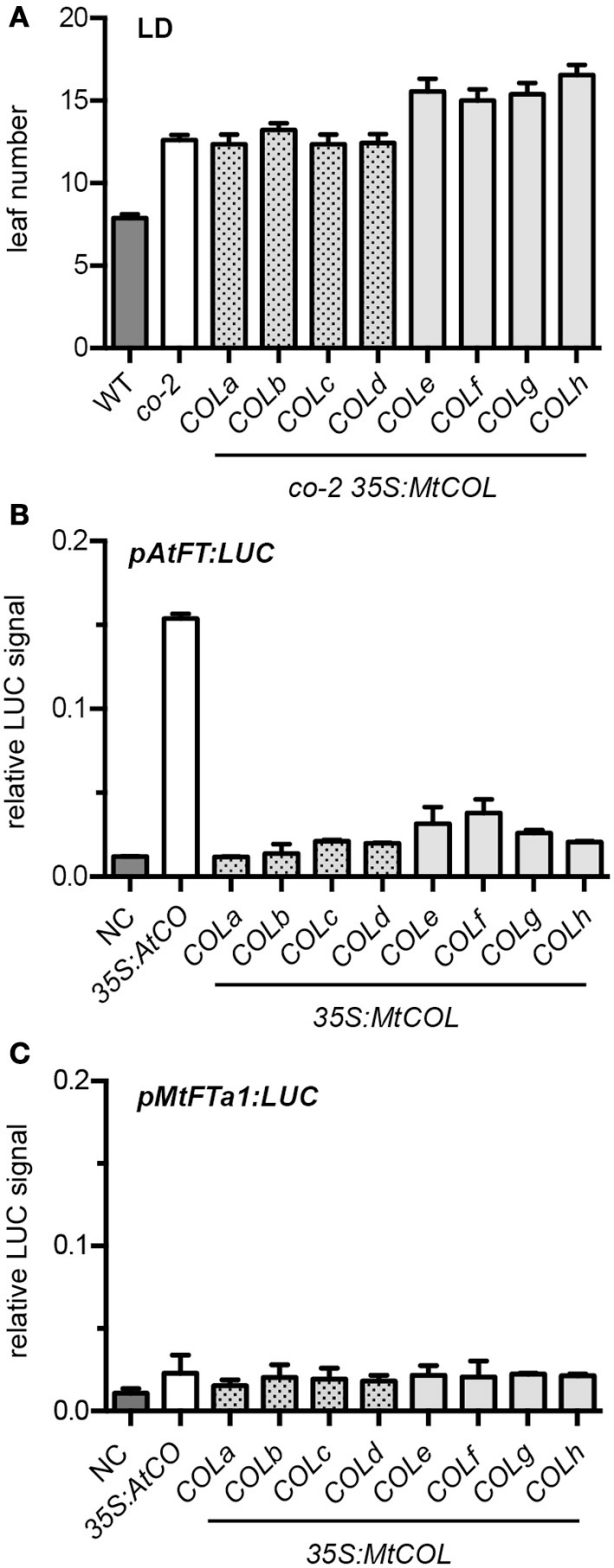
**Functional analyses of *MtCOL* genes. (A)**. Overexpression of *MtCOLa-COLh* genes does not promote flowering in the Arabidopsis *co-2* mutant. Flowering time is indicated by leaf number at flowering. Data represent a minimum of 10 plants for each line ± SE. **(B)**. *MtCOL* genes are unable to induce expression from the Arabidopsis *FT* promoter in transient expression assays. The *35S:AtCO* construct and *35S:MtCOL* constructs were co-infiltrated with *AtFT* promoter fused to the luciferase (LUC) reporter gene into *N. benthamiana* leaves. Only *35S:AtCO* and *35S:MtCOLf* resulted in statistically significant upregulation of the *AtCO* promoter compared with the NC (no construct) control, *P* < 0.0001 and *P* = 0.045, respectively **(C)**. *MtCOL* genes are unable to induce expression from the Medicago *FTa1* promoter in transient expression assays. The *35S:AtCO* construct and *35S:MtCOLa-h* constructs were co-infiltrated with *MtFTa1* promoter:LUC into *N. benthamiana* leaves. No statistically-significant difference in relative LUC signal between the NC control and *35S:AtCO* or the *35S:MtCOLs* was observed. NC (no construct) refers to leaves infiltrated with untransformed *Agrobacterium* along with the *AtFT* or *MtFTa1* promoter:LUC constructs. Relative LUC signal is a ratio of LUC activity versus Renilla luciferase activity to correct for variation in transformation efficiencies between infiltrated *N. benthamiana* leaves and data represent the mean ± SE of three biological replicates. Statistical analysis was performed using Student's *t*-test. In all panels, Group I and Group III *MtCOL* genes are represented by dark gray and light gray shading, respectively.

Arabidopsis CO is a potent inducer of flowering, and Arabidopsis plants overexpressing *CO* flower very early under both LD and SD (Onouchi et al., 2000). To test whether any of the *MtCOL* genes might be similarly effective in flowering regulation, we assessed their ability to complement the late flowering phenotype of the Arabidopsis *co-2* mutant. Figure 3A shows that none of the eight *MtCOL* genes that we tested caused early flowering when overexpressed from the cauliflower mosaic virus 35S promoter in the late-flowering Arabidopsis *co*-2 mutant plants.

Next, we examined the ability of the *MtCOL* genes to directly activate the *Arabidopsis FT* promoter using a transient assay system. In this system, the *Arabidopsis FT* promoter was fused to the luciferase reporter gene and infiltrated into *Nicotiana benthamiana* leaves together with different transcription factors. Figure 3B shows that expression of *AtCO* resulted in substantial upregulation of luciferase expression from the *Arabidopsis FT* promoter (*P* < 0.0001). In contrast, the majority of *MtCOL* genes had no clear statistically significant effect consistent with their inability to complement the *co-2* mutant. The one possible exception was *MtCOLf*, which showed a small increase in LUC signal with marginal statistical significance (*P* = 0.045).

The transient assay system was also used to investigate if any of the *MtCOL* genes are able to activate the *Medicago FTa1* promoter. The *Medicago FTa1* gene plays a key role in promoting flowering in response to both vernalization and LD (Laurie et al., 2011). When *Medicago* plants are shifted from SD to LD, *FTa1* is upregulated by exposure to a single long day (Laurie et al., 2011). An *MtFTa1* promoter sequence comprising 2017 bp upstream of the start codon was fused to the luciferase reporter gene and infiltrated into *Nicotiana benthamiana* leaves together with different *MtCOL* genes. Neither Arabidopsis *CO* nor any of the *MtCOL* genes was able to induce *LUC* expression from this promoter sequence (*P* > 0.05 in all cases) (Figure 3C).

Overall, these results provide further evidence that that none of the *MtCOL* genes are functionally equivalent to *AtCO*, with respect to their ability to induce expression of *AtFT*. In addition they also suggest that neither *AtCO* nor any of the tested *MtCOL* genes are able to induce *MtFTa1* expression. Although the specific reason for the inactivity of *MtCOL* genes on *AtFT*, and *AtCO* on *MtFTa1* is not yet clear, it could partially reflect divergence in FT promoter sequences and/or DNA binding characteristics of CO and COL proteins. An alignment of the *AtFT* proximal promoter with regions upstream of the transcriptional start site in the Medicago and chickpea *FTa1* genes (Supplemental Figure 3) shows that neither of the two CO-responsive (CORE) elements defined in the AtFT promoter are significantly conserved in the legume promoters, which may provide an explanation for the inactivity of AtCO on the *MtFTa1* promoter.

### Genetic analysis of *COL* function

**Figure 4:**
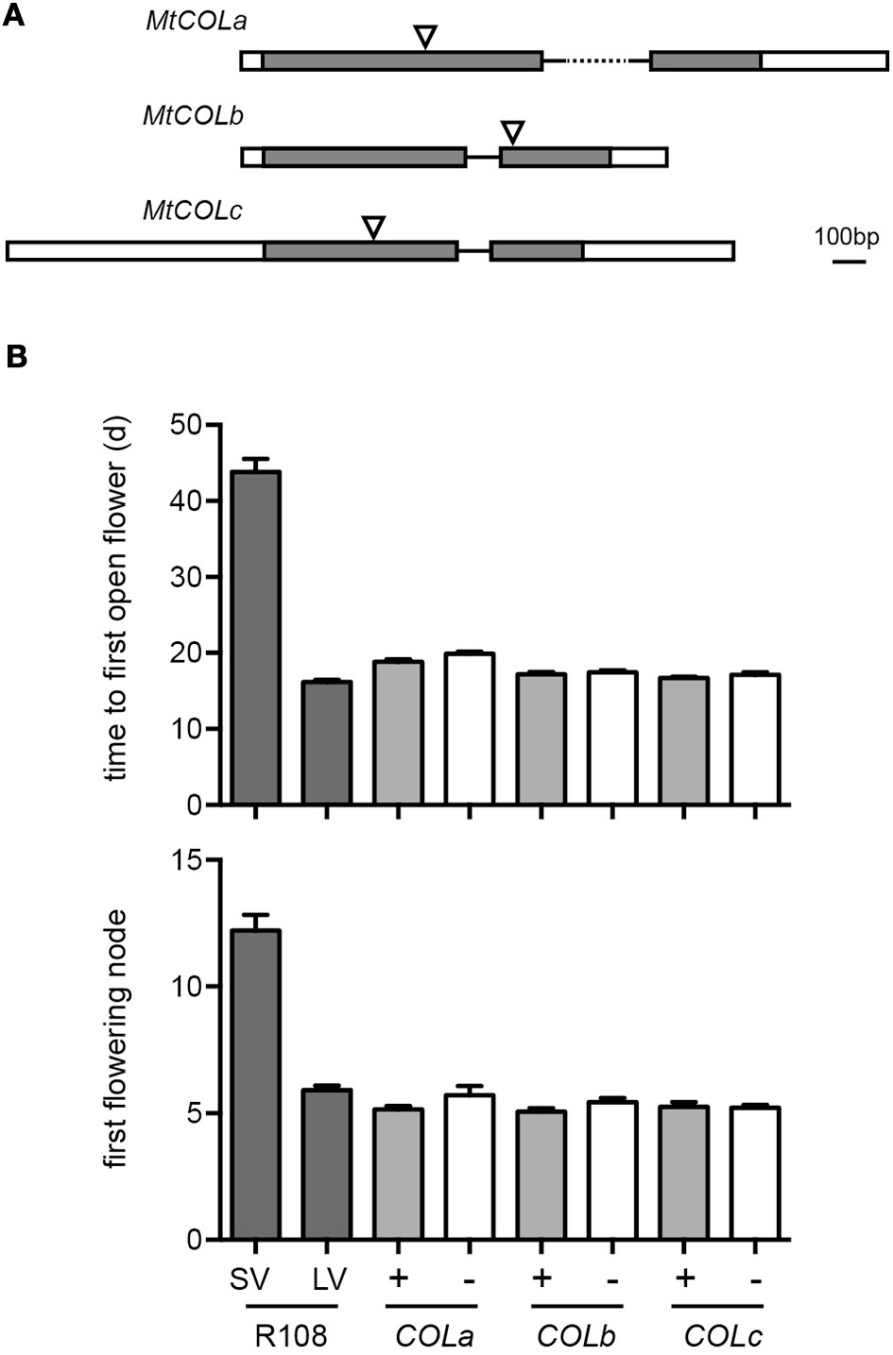
**Characterisation of mutants for *MtCOLa, COLb* and *COLc*. (A)** Diagram of *MtCOLa, COLb* and *COLc* genes showing gene structure and site of the *Tnt1* insertion. Exons are shown as boxes with the coding sequence in dark gray, and the 5′ and 3′ untranslated regions in white. **(B)** Flowering time and node of first flower for WT and *COL* mutant plants vernalized for 14 days at 4°C before transfer to LD at 22°C. Homozygous WT (+) and mutant (−) genotypes were selected from segregating progenies and are represented by by shaded and empty bars, respectively. Vernalized R108 plants grown under LD (LV) or SD (SV) were included as a control. Values represent mean ± SE for *n* = 12–20.

Finally, in order to directly examine *COL* gene function, we made use of the Medicago *Tnt1* insertion platform (Tadege et al., 2008) to identify putative insertion mutants for three of the four Group I *MtCOL* genes. Insertions in *COLa*, *COLb* and *COLc* were verified by sequencing and mutant lines shown to specifically lack the corresponding transcript (Figure 4A). For phenotypic comparisons we vernalized seeds for 2 weeks at 4°C and grew seedlings under an 18 h photoperiod. In addition to a pure line of the progenitor line R108, we also included WT lines selected from individual segregating progenies for each mutant as controls. Figure 4B shows that neither *colb* nor *colc* mutants flowered significantly differently from their corresponding control lines in terms of either flowering time (*P* > 0.5 and *P* > 0.2 for *colb* and *colc*, respectively) or for node of first flower (*P* = 0.088 and *P* > 0.5, respectively). The genetic background carrying the *cola* mutation was slightly later flowering than the R108 control line, in terms of days (18.9 vs. 16.2 days, *P* < 0.001), but slightly earlier in terms of nodes (5.2 vs. 5.9 nodes, *P* < 0.01). The *cola* mutant line was marginally later than its WT control line for both time (19.9 vs. 18.9 days, *P* = 0.044) but not for node number (5.7 vs. 5.2 nodes, *P* = 0.088). However, importantly, the variation in flowering time or node observed within and between these lines was negligible relative to the strong delay of flowering in vernalized R108 plants under SD, indicating that none of these three *COL* genes contributes significantly to the promotion of flowering by LD.

## Discussion

The CONSTANS protein has been a central feature of models explaining the molecular basis for plant responses to photoperiod, and the potential conservation of CO function across flowering plants has been a topic of considerable interest. In legumes, several studies have identified conserved elements of the photoperiod response pathway, including homologs of *GIGANTEA* (Hecht et al., 2007; Watanabe et al., 2011), *PHYA* (Weller et al., 2004; Liu et al., 2008a; Watanabe et al., 2009), *FT* (Kong et al., 2010; Hecht et al., 2011; Laurie et al., 2011; Sun et al., 2011) and circadian clock genes (Liew et al., 2009, 2014; Weller et al., 2012), but the potential role of *CO*-like genes has received less attention. In this study we have identified 11 *COL* genes in the model long-day legume *Medicago truncatula*, and investigated the regulation and function of eight of these. Collectively our results provide several strong lines of evidence that the three genes most similar to Arabidopsis *CO*, *MtCOLa, MtCOLb* and *MtCOLc*, genes do not participate in the induction of flowering by photoperiod. Our results also indicate that the five other genes we examined (*MtCOLd-COLh*) are also unlikely to function in a manner similar to *AtCO*.

The first line of evidence comes from regulation of *MtCOL* expression. Rhythmic expression of group I and group III *MtCOL* genes showed broad similarity to reported results from other species, with group I genes showing peak expression around dawn and other genes generally more strongly expressed late in the day or in the early part of the night (Figure 2). However, with the possible exception of *MtCOLf*, we found no evidence for photoperiod-specific coincidence of *MtCOL* expression with the light phase in LD, or for the afternoon peak that is characteristic of the *AtCO* transcriptional rhythm under LD (Imaizumi et al., 2003) (Figure 2). Nevertheless, it should be noted that the absence of these regulatory features does not in itself exclude the possibility that these genes have *CO*-like function. First, because Arabidopsis *CO* is known to undergo significant post-transcriptional regulation, and it is conceivable that photoperiod-specific activity could be conferred on one or more of the *MtCOL* genes predominantly through regulation at the protein level. Second, because the link between *CO* expression dynamics and photoperiod responsiveness has only been elaborated in detail for Arabidopsis and it is not yet clear how widely this may be conserved (Ballerini and Kramer, 2011).

We obtained more direct evidence on *MtCOL* functions from Arabidopsis complementation and experiments using a tobacco transient assay system. None of the *MtCOL* genes was able to promote flowering when overexpressed in the Arabidopsis *co-2* mutant (Figure 3A), in contrast to the strong flower promoting activity of Arabidopsis *CO* (Onouchi et al., 2000). This contrast was also seen in transient assays, where none of the eight tested *MtCOL* genes was able to activate transcription from the Arabidopsis *FT* promoter, even though *AtCO* clearly possessed this ability (Figure 3B). Finally, transcript-null mutants for the Group Ia gene *MtCOLa* and two other Group I genes all flowered normally under LD after vernalization (Figure 4), clearly indicating that these genes are not needed for, and likely do not participate in, the promotion of flowering by LD.

Overall, the lack of any clear effect of *MtCOL* genes on flowering is somewhat surprising, particularly in the case of *COLa*, in view of the fact that Group Ia *COL* genes across a range of species have been shown to some degree of function in flowering regulation. Outside of Arabidopsis, the involvement of Group Ia *COL* genes in photoperiod responsiveness is most conclusive in rice, where the single group Ia *COL* gene *Hd1* underlies a major-effect QTL for flowering, and has a bidirectional role in regulation of *FT* homologs (Yano et al., 2000). The potato CO gene also has a clear endogenous role in photoperiod responsiveness, although this is less pronounced for flowering induction than for tuberization (Navarro et al., 2011). Group Ia *COL* genes in a number of other species have shown flower-promoting activity in Arabidopsis. Genes from potato, tomato, poplar and sugar beet are able to at least partially complement Arabidopsis *co* mutants (Ben-Naim et al., 2006; Chia et al., 2008; Gonzalez-Schain et al., 2012; Hsu et al., 2012). The barley *Hd1* ortholog *CO1* has no activity in transgenic Arabidopsis but does promote flowering when overexpressed in barley itself (Campoli et al., 2012).

In Arabidopsis, activation of the Arabidopsis *FT* promoter by CO requires several regulatory motifs located within 500 bp of the *FT* transcriptional start site (Adrian et al., 2010; Tiwari et al., 2010). Full *FT* activation in planta requires the additional association of CO with proteins bound to distal promoter regions and the formation of chromatin loops (Ben-Naim et al., 2006; Adrian et al., 2010; Cao et al., 2014). However, a 1 kb proximal fragment of the *AtFT* promoter is sufficient for maximal induction by *AtCO* in a transient assay system (Adrian et al., 2010) and our results show that *MtCOL* genes lack this activity, consistent with their lack of flower-promoting activity when overexpressed in Arabidopsis (Figure 4A). While the reason for this is not clear, the simplest explanation may be that *MtCOL* proteins either do not bind to the CO-responsive elements in this region, or simply do not function as transcriptional regulators. Our results also show that an equivalent region of the *MtFTa1* promoter is activated neither by *AtCO* nor *MtCOL* genes. The lack of *AtCO* activity may reflect the fact that none of the functionally validated proximal elements in the Arabidopsis *FT* promoter are significantly conserved in the corresponding regions of the Medicago or chickpea *FTa1* genes (Supplemental Figure 3).

The argument may also be made that *CO* function may be preserved in the *MtCOL* family but is comprised of small individual contributions from multiple members. Clearly the present data also do not exclude this possibility, with some features of *MtCOLf* (Figures 2, 3B) consistent with a weak CO-like effect. However, it is worth noting that loss-of-function variants for Arabidopsis *CO* and rice *Hd1* both have large phenotypic effects and were first identified through relatively direct forward genetic analysis. This is not proof that deep redundancy within the *COL* family is not an explanation for our results, but does make it seem less likely. It also remains possible that *CO* function could be carried out one or more of the other *MtCOL* genes that we did not examine, which include both group II (*COLi*, *COLk*) and Group III (*COLj*) genes. Although no gene outside the group Ia *COL* clade has been implicated in photoperiodic flowering, a more general effect on flowering has been demonstrated for certain other *COL* genes. In Arabidopsis, the group Ic gene *COL3* (Datta et al., 2006) and the group III gene *COL9* (Cheng and Wang, 2005) both inhibit flowering, whereas a second group Ic gene, *COL5*, may have a promotive function (Hassidim et al., 2009). In rice, the Group Ic gene *COL4* gene inhibits flowering under SD and LD through repression of *FT* homologs *RFT* and *Hd3a* (Lee et al., 2010).

Recently, new information has emerged on *COL* genes in the short-day legume soybean. Soybean has multiple group Ia *COL* genes, which comprise two pairs of homeologs; *COL1a/b* and *COL2a/b*. Two recent studies show that one of these, *GmCOL2a*, is able to complement the Arabidopsis *co-2* mutant (Fan et al., 2014; Wu et al., 2014), and there is evidence that the remaining genes *COL1a/b* and *COL2b* may also have some activity in Arabidopsis (Wu et al., 2014). However, our phylogenetic comparisons indicate that Medicago has only a single Group Ia *COL* gene orthologous to Arabidopsis *CO*/*COL1*/*COL2*, and does not have an ortholog of the *GmCOL2* genes (Supplemental Figure 2). Sequence searches in other temperate legumes (including pea, and chickpea and *Lotus japonicus*) also identify only a single group *Ia COL* gene in these species, indicating that loss of *COL2* orthologs may have occurred relatively early in this temperate legume lineage.

Overall, it seems on balance likely that *COL* genes do not function as central integrators of photoperiod responsive flowering in Medicago. This may also be more generally true across the temperate legumes, at least for *COLa*, as *COLa* orthologs in pea and *Lotus japonicus* also do not show characteristic regulatory features of Arabidopsis *CO* (Hecht et al., 2007; Yamashino et al., 2013). Instead, *CO*-independent pathways may have a more prominent role in this plant group. In Arabidopsis, a number of other factors contribute to direct regulation of *FT* expression. These include the positive factors PIF4 and CIB1, bHLH proteins involved in light signaling, and SPL3, which is a target of the *miRNA156* pathway controlling juvenility (Liu et al., 2008b; Kim et al., 2012; Kumar et al., 2012). Factors repressing *FT* include the CDF family of Dof transcription factors, and a number of AP2 domain proteins that are targets of *miR172* (Jung et al., 2007; Fornara et al., 2009). In particular, it is intriguing that despite the apparent absence of *CO*-like function in Medicago, evidence from the related legume pea shows a major role for the *GI* ortholog *LATE1* (Hecht et al., 2007, 2011). In Arabidopsis *GI* has been shown to promote *FT* transcription independently of *CO*, both by contributing to degradation of CDF proteins (Song et al., 2012), and also by positive effects on miR172 biogenesis (Jung et al., 2007). In addition, the recently-identified B3-transcription factor-like gene *E1* in soybean is also important for photoperiod-dependent regulation of *FT* expression, and an ortholog is present in Medicago (Xia et al., 2012; Zhai et al., 2014). Whether one or more of these mechanisms contribute to the photoperiod response in temperate legumes will be an important question for future investigation.

### Conflict of interest statement

The authors declare that the research was conducted in the absence of any commercial or financial relationships that could be construed as a potential conflict of interest.
