# Supplementary material for: Isolation and functional analysis of CONSTANS-LIKE genes suggests that a central role for CONSTANS in flowering time control is not evolutionarily conserved in Medicago truncatula
Source: Front Plant Sci. 2014 Sep 18;5:486. doi: 10.3389/fpls.2014.00486 (PMC4166892; doi:10.3389/fpls.2014.00486)
Supplement: Supplementary file 1 [file DataSheet1.PDF]

Supplemental Figure 1. Alignment of CONSTANS-like proteins from Medicago, soybean and Arabidopsis. Sequences were aligned using Clustal X and manually adjusted. Shading levels indicate degree of conservation – black = 100%, dark grey = 80%, light grey = 60%. The location of conserved B-Box and CCT domains are indicated. Details of legume sequences are given in **Supplemental Table 1.** The identity and nomenclature for Arabidopsis COL genes follows Griffiths et al (2003).

```
                     *        20         *        40         *        60         *        80         *       100         *       120         *       140         *
AtCO      : ----------------------------MLKQ------ESNDIGSGENNRARPCDTCRSNACTVYCHADSAYLCMSCDAQVHSANRVASRHKRVRVCESCERAPAAFLCEADDASLCTACDSEVHSANP---------LARRHQRVPILP : 107
AtCOL2    : ----------------------------MLKE------ESNESGT----WARACDTCRSAACTVYCEADSAYLCTTCDARVHAANRVASRHERVRVCQSCESAPAAFLCKADAASLCTACDAEIHSANP---------LARRHQRVPILP : 103
AtCOL1    : ----------------------------MLKV------ESN--------WAQACDTCRSAACTVYCRADSAYLCSSCDAQVHAANRLASRHERVRVCQSCERAPAAFFCKADAASLCTTCDSEIHSANP---------LARRHQRVPILP :  99
GmCOL1a   : ----------------------------MLDG-----------EATMGTWARMCDTCRSAPSSVFCRAHTAFLCATCDARLHAS---LTWHERVWVCEACERAPAAFLCKADAASLCASCDADIHAANP---------LASRHHRVPILP :  99
GmCOL1b   : ----------------------------MLEG-----------QATTPTWPRMCDTCRSVPSTVFCRSHTAFLCATCDTRLHVS---LTWHERVWVCEACERAPAAFLCKADAASLCASCDADIHAANP---------LASRHHRVPILP :  99
MtCOLa    : ----------------------------MLEQDFLTTTSATATVRSAGTWARTCDTCRSAPCAVFCRADSAYLCAACDARIHAANRVASRHERVWVCEACERAPAAFLCKADAASLCSTCDADIHSANP---------LASRHQRVPILP : 113
GmCOL2a   : ----------------------------MLKEG----TNNVGGS-TG-TWSHVCDTCRSAPCVLYCHADSAYLCSSCDARVHAANRVASRHERVWVCEACERAPAAFLCKADAASLCSSCDADIHSANP---------LASRHHRVPILP : 107
GmCOL2b   : ----------------------------MLKEG----TNNVGGSNTGTTWSRVCDTCLSAPCVLYCHADSAYLCSSCDARVHAANRVASRHKRVWVCEACERAPAAFLCKADAASLCSSCDADIHSANP---------LASRHNRVPILP : 109
GmCOL3a   : -----------------------------------------------MASK-LCDSCKSATATLYCRPDAAFLCGACDSKVHAANKLASRHPRVALCEVCEQAPAHVTCKADAAALCLACDRDIHSANP---------LASRHERIPVSP :  93
GmCOL3b   : -----------------------------------------------MASK-LCDSCKSATATLYCRPDAAFLCGACDSKVHAANKLASRHPRVVLCEVCEQAPAHVTCKADAAALCLACDRDIHSANP---------LASRHERIPVTP :  93
MtCOLc    : -----------------------------------------------MASK-LCDSCKSATATLYCRPDSAFLCGACDSKVHAANKLASRHPRVTLCEVCEQAPAHVTCKADAASLCITCDRDIHTANP---------LAARHERVPVTP :  93
MtCOLb    : -----------------------------------------------MATK-LCDSCKSTKATLFCRSDSAFLCLTCDSNIHAANKLASRHHRVTLCQVCEQAPAHVTCKADAAVLCISCDHDIHSANP---------LARRHERVPLTT :  93
AtCOL3    : ---------------------------------------------MASSSR-LCDSCKSTAATLFCRADAAFLCGDCDGKIHTANKLASRHERVWLCEVCEQAPAHVTCKADAAALCVTCDRDIHSANP---------LSRRHERVPITP :  95
AtCOL4    : MDPTWIDSLTRSCEANSNTNHKRKRERETLKHR---EKKKKRFRERKMASK-LCDSCKSATAALYCRPDAAFLCLSCDSKVHAANKLASRHARVWMCEVCEQAPAHVTCKADAAALCVTCDRDIHSANP---------LARRHERVPVTP : 137
GmCOL4a   : ----------------------------MGIER---GGLKGFRSGWSVPPKKPCDSCKLASAALFCHLDSAFLCIACDSKIHCANKLASRHERVWMCEVCEQAPASVTCKADAAALCVTCDSDIHSANP---------LAQRHERVPVEP : 110
GmCOL4b   : ----------------------------MGIER---GGFKGFRSAWSVPPK-PCDSCKLASAALFCRPDSAFLCIACDSNIHCSNKLASRHERVWMCEVCEQAPAAVTCKADAAALCVTCDSDIHSANP---------LAQRHERVPVEP : 109
MtCOLd    : ----------------------------MGIER---GGLKSLRGGWSVPPK-LCDSCKLTPAALFCRSDSAFLCINCDSTIHSANKLSSRHERVWMCEVCEQAPASVTCKADAAALCVTCDSDIHSANP---------LARRHERVPVEP : 109
AtCOL5    : ----------------------------MGFGL---ESIKSISGGWGAAAR-SCDACKSVTAAVFCRVDSAFLCIACDTRIHS----FTRHERVWVCEVCEQAPAAVTCKADAAALCVSCDADIHSANP---------LASRHERVPVET : 105
AtCOL9    : -------------------------------------------------MGYMCDFCGEQRSMVYCRSDAACLCLSCDRSVHSANALSKRHSRTLVCERCNAQPATVRCVEERVSLCQNCDWSGHNNSNNNNSSSSSTSPQQHKRQTISC : 101
AtCOL10   : -------------------------------------------------MGYMCDFCGEQRSMVYCRSDAACLCLSCDRNVHSANALSKRHSRTLVCERCNAQPASVRCSDERVSLCQNCDWSGHDGKN-------STTTSHHKRQTINC :  94
GmCOL8a   : -------------------------------------------------MGYLCDFCGDQRSLVYCRSDSACLCLSCDRNVHSANALSRRHSRTLVCERCNSQPAFVRSVEEKISLCQNCDWLGHGTSP---------SSSMHKRQSINC :  92
GmCOL8b   : -------------------------------------------------MGYLCDFCGDQRSLVYCRSDAACLCLSCDRNVHSANALSRRHSRTLVCERCNSQPAFVRCVDEKISLCQNCDWLGHGTSP---------SSSTHKRQSINC :  92
GmCOL9a   : -------------------------------------------------MGYICDFCGDQRSMVYCRSDAACLCLSCDRNVHSANALSKRHSRTLLCERCNSQPAFVRCVEEKISLCQNCDWLGHGTST---------SSSTHKRQAINC :  92
GmCOL9b   : -------------------------------------------------MGYICDFCGDQRSMVYCRSDAACLCLSCDRNVHSANALSKRHSRTLLCERCNSQPAFVRCVEEKISLCQNCDWLGHGTST---------SSSTHKRQAINC :  92
MtCOLf    : -------------------------------------------------MGSLCDFCGDQRSLVYCRSDAASLCLSCDRNVHSANELSKRHSRTLVCERCNLQPAYVRCVEEKVSLCQNCDWSAHGTNP---------SSSTHKRQSINC :  92
MtCOLe    : -------------------------------------------------MGYICDFCGDQRSMVYCRSDAACLCLSCDRNVHSANTLARRHSRTLLCERCSSQPALVRCSEEKVSLCQNCDWLGHGNS----------TSSNHKRQTINC :  91
AtCOL11   : -------------------------------------------------MEARCDFCGTEKALIYCKSDSAKLCLNCDVNVHSANPLSQRHTRSLLCEKCSLQPTAVHCMNENVSLCQGCQWTASNCTG-----------LGHRLQSLNP :  90
AtCOL12   : -------------------------------------------------MEPKCDHCATSQALIYCKSDLAKLCLNCDVHVHSANPLSHRHIRSLICEKCFSQPAAIRCLDEKVSYCQGCHWHESNCSE-----------LGHRVQSLNP :  90
GmCOL10a  : -------------------------------------------------MDPLCEFCGVVRAVVYCKSDSARLCLHCDGCVHSANSLSRRHSRSLLCDKCNSQPAMIRCMDHKLSLCQGCDWNPNDCSA-----------LGHRRVALNC :  90
GmCOL10b  : -------------------------------------------------MDPLCEFCRVVRAVVYCKSDSARLCLHCDGCVHSANSLSRRHSRSLLCDKCNSQPAMIRCMNHKLSLCQGCDWNPNDCSA-----------LGHRRLALNC :  90
MtCOLj    : -------------------------------------------------MEALCEFCGVARAVVYCKPDSARLCLHCDGNVHSANSLSRRHPRSLLCDKCNFDSAIVRCVDHKLSLCQVCDWNTNDCFV-----------LGHKHVLLTF :  90
GmCOL11a  : ---------------------------------------KQKAIAMSG-EARSCDYCGHSTALLYCRADSAKLCFSCDREVHSTNQLFSKHTRTLLCDACDHSPATILCSTDTSVLCQNCDWEKHN---------PALSDSLHERRPLEG : 101
GmCOL11b  : ---------------------------------------------MSGAEARPCDYCGNSTALLYCRADSAKLCFSCDREVHSTNQLFSKHTRTLLCDACDDSPATILCSTDTSVLCQNCDWENHN---------PALSDSLHERRPLEG :  96
MtCOLg    : ----------------------------------MGGSPRNPNPNPSHKLVRPCDYCGHSNAVIYCRADSAKLCFSCDREVHSTNQLFSKHTRSLICDSCDDSPATILCSTESSVFCQNCDWENHN---------LSLS-SPHERRSLEG : 106
GmCOL12a  : ---------------------------------------MMNGSP--NSKQRTCDYCGSFTALLYCRADSAKLCFFCDRKVHFPNQLFSKHKRAQLCDACGDSPASVLCSAENSVLCQNCDCGKQK---------HLVS-EAHQRRPLEG :  99
GmCOL12b  : ---------------------------------------MMSGSPSPNSKQRTCDYCGDFTALLYCSADSAKLCFFCDRKVHSPNQLFSKHTRAQLCDSCGDSPASVLCSAENSVLCHNCDCEKHK---------HLAS-EVHQRKPLEG : 101
AtCOL13   : -----------------------------------------MEAEEGHQRDRLCDYCDSSVALVYCKADSAKLCLACDKQVHVANQLFAKHFRSLLCDSCNESPSSLFCETERSVLCQNCDWQHHT---------ASSS--LHSRRPFEG :  98
AtCOL14   : ------------------------------------------MGTSTTESVVACEFCGERTAVLFCRADTAKLCLPCDQHVHSANLLSRKHVRSQICDNCSKEPVSVRCFTDNLVLCQECDWDVHG------SCSSS---ATHERSAVEG :  99
AtCOL15   : --------------------------------------------MSSSE-RVPCDFCGERTAVLFCRADTAKLCLPCDQQVHTANLLSRKHVRSQICDNCGNEPVSVRCFTDNLILCQECDWDVHG------SCSVS---DAHVRSAVEG :  96
GmCOL13a  : --------------------------------------------------MLPCDYCHSKPAILFCRPDSAKLCLLCDQHVHAANALSLKHVRFQICDSCKTDTAVLRCSTDNLVLCHHCDVETHG------AAASS----HHQRHRLHG :  90
GmCOL13b  : --------------------------------------------------MLPCDYCDSKPALLFCRADSAKLCLVCDQHVHAANALSLKHVRFQICDSCKSDTAVLRCSTHNLVLCHNCDVDAHG------ADASSLHHHHHHHRRLHG :  94
MtCOLh    : -------------------------------------------------MSFPCDYCDTRSAVLYCKPDSAKLCLVCDQHVHSANALALKHVRFQICQNCKNDAASVRCFTENLVQCHRCDWNSHGGDD---DDSTSSSFHHHNRRRIEG :  98
AtCOL6    : ----------------------------------------------------------------------------------------------------------------SPPHQ-ATWHQGFTRKA------------RTPRG-GKK :  24
AtCOL16   : -----------------------------------MMKS--LANAVGAKTARACDSCVKRRARWYCAADDAFLCQSCDSLVHSANPLARRHERVRLKTASPAVVKHSNHSSASPPHEVATWHHGFTRKA------------RTPRGSGKK : 101
GmCOL7a   : --------------------------------MSSATKN--AANAVGAKTARACDSCITKRARWYCAADDAFLCQACDSSVHSANPLARRHERVRLKTAS------YKSTDEQQQQQQQQPPTWHTKKP------------RTPRHGKHS :  98
GmCOL7b   : --------------------------------MSSATKN--AANAVGGKTARACDGCITKRARWYCAADDAFLCQACDSSVHLANPLARRHERVRLKTAS------YKSTDER-----RQPPTWHTKKP------------RTPRHGKHS :  93
MtCOLk    : --------------------------------MTCSSKN--VANAVGGKTARACDSCITKRARWYCAADDAFLCQACDSSVHSANSLARRHERVRLKTAS------YKSINGDEFFN-CGPFSGFTKKA------------RTPRQGKHK :  97
GmCOL6a   : ---------------------------------MRDMK---DAGALGGKTARACDSCVSRRARWFCAADDAFLCHGCDTLVHSANQLASRHERVRLQTAS--------SKV--TTT--HAWHSGFTRKA------------RTPRHN--- :  87
GmCOL6b   : ---------------------------------MRDMK---DAGALGGKTARACDSCVSRRARWFCAADDAFLCHACDTLVHSANQLASRHERVRLQTAS--------SKA--TTTTTHAWHSGFTRKA------------RTPRHNN-- :  90
MtCOLi    : --------------------------------MIIDMKGDADAGALGAKTARACDSCLRRRARWFCAADDAFLCHGCDNLVHSANLLASRHERVRLQTAS--------AKV--TTT-AQAWHSGFTRKA------------RTPRHNK-- :  93
GmCOL5a   : --------------------------------MTNEMK---EASALGARTARACESCLKVRARWYCAADDAFLCHGCDNMVHSANQLASRHERVKLQTAS--------SKVNYSVTPKVAWHSGFTRKA------------RTPRHNNNR :  95
GmCOL5b   : --------------------------------MTNEMK---EASALGARTARACESCLKVRARWYCAADDAFLCHGCDNMVHSANQLASRHERVKLQTAS--------SKVNYSVTPKVAWHSGFTRKA------------RTPRSNN-K :  94
AtCOL7    : -------------------------------------------------------------------------------------------TETADKTTS-------------------VWYEGFRRKA------------RTPRSKSCA :  28
AtCOL8    : --------------------------MTSHQNIKISEKIMISKYQEDVKQPRACELCLNKHAVWYCASDDAFLCHVCDESVHSANHVATKHERVCLRTNEIS------NDVRGGTTLTSVWHSGFRRKA------------RTPRSRYEK : 106
```

B-box 1

B-box 2 / diverged B-box

```
                   160        *        180        *        200        *        220        *        240        *        260        *        280        *        300
AtCO     : ISGNSFSSMTTTHHQSEKTMT--------------------------------------DPEKRLVVDQEEG-EEGDKDAKEVASWLFPNSDKN-------------------------------------------------------- : 162
AtCOL2   : LSANSCSSMAPSETDAD----------------------------------------------------------NDEDDREVASWLLPNPGKN-------------------------------------------------------- : 139
AtCOL1   : ISEYSYSS-TATNHSCETTVT--------------------------------------DPENRLVLGQ----EEEDEDEAEAASWLLPNSGKN-------------------------------------------------------- : 150
GmCOL1a  : IA-----------------------------------------------------------AAPGNNDNDNVDDADLDDDDETASWLLLNPVKS-------------------------------------------------ASVP--N : 139
GmCOL1b  : IA-----------------------------------------------------------AAN-NNNNDDDDVADVDDEDETASWLLLNPIKS-------------------------------------------------ATVPNTN : 140
MtCOLa   : ISGYLYGPPATLLGAEDEGFV--------------------------------------RGGCEVEEEEDEGVDHDMEDENEAASWLLLNPLKNNNNNSN-------------------------------------------NNISNDH : 182
GmCOL2a  : ISGSLFGEPE--HER---VYA--------------------------------------FVNEVEAEEEEEEVFDEY-DEVEAASWLLPHPMKN---------------------------------------------------DKIDE : 162
GmCOL2b  : ISGSLFREPE--HNHKRVEHA--------------------------------------FVNEVE--EEEEGVFDEYEDEVEAASWLLPHPMKNN--------------------------------------------------DEIEE : 167
GmCOL3a  : FFES--VHSVKAS----SPIN--------------------------------------FLDDHRFFS--DADADVSTEEAEAASWLLPNPK--------------------------------------------------------TD : 141
GmCOL3b  : FFES--VHSVKAS----SPIN--------------------------------------FH--HRFFSDADADADVSTEEAEAASWLLPNPK--------------------------------------------------------TD : 141
MtCOLc   : FFESNTSHSVKSL----NN--------------------------------------------NN------NNYDAVKDEAEAASWLISDPK--------------------------------------------------------AD : 133
MtCOLb   : TFNHQNSQQQSFF----SE----------------------------------------------------NDHDATTEEAEAASWLLQTPSNPK----------------------------------------------------FPD : 135
AtCOL3   : FYDAVGPAKSAS-----SSVN--------------------------------------FVD--------------EDGGDVTASWLLAKEG--------------------------------------------------------IE : 132
AtCOL4   : FYDSVSSDGSVKH----TAVN--------------------------------------FLDDCYFSD-IDGNGSREEEEEEEAASWLLLPNPKTTTTA---------------------------------------------TAGIVA : 198
GmCOL4a  : FFDSAESIVKAS-----ATAS--------------------------------------FGFVVPSDDGAASDV-FAPDDSDSAAWLIPNPNFGS-----------------------------------------------------KL : 163
GmCOL4b  : FFDSAESIVKAS-----AAAT--------------------------------------FGFIVPSDDGGASDA-FAPDDSDAAAWLIPNPNFGS-----------------------------------------------------KL : 162
MtCOLd   : FFDSAESVVKSSSAAAAAAAS--------------------------------------FNFVVPTDDG------YGQDDAEAAAWLIPNPNFGS-----------------------------------------------------KL : 162
AtCOL5   : FFDSAETAVAKIS----ASST--------------------------------------FGILGSSTTVDLTAVPVMADDLGLCPWLLPN-DFNEPA--------------------------------------------------KIE : 162
AtCOL9   : YSGCPSSSELASIWSFCLDL-------------------------------AGQSICEQELGMMNIDDD---------------GPTDKK-----TCNEDKKD------VLVGSS------SIP--ETSSVPQG-------KSSSAKDVG : 179
AtCOL10  : YSGCPSSAELSSIWSFCMDLNIS---------------------------SAEESACEQGMGLMTIDED---------------GTGEKS-----GVQKINVE------QPETSS------AAQGMDHSSVPE--------NSSMAKELG : 177
GmCOL8a  : YSGCPSAAEFSSIWSFFLDIP-----------------------------SLGEA-CEQELGLMSINEN------IPPEGQNVSGSTEVT-----DLPSKGKSWAGTPSIPESSSEPRILDQPPGPANECVPKL-------YCPGKKASG : 194
GmCOL8b  : YSGCPSAAEFSSIWSFFLDIP-----------------------------SMGEA-CEQELGLMSINENSNKNAWASPEGQNVSGSAEVT-----DLPSKGKSWAGTSSVPESSSEPRILDQPPGPANECMPKL-------YCPGKKVSG : 200
GmCOL9a  : YSGCPSAAELSSIWSFVLDIA-----------------------------AISESTCEQELGLMSINEN--KSVGVPPEGQNVSGSDEVT-----DQPALDKSLVGTSSMPESSSKPRILDQPARPANECLSKL-------YCPATKFPA : 199
GmCOL9b  : YSGCPSAAELSSIWSFVLEIA-----------------------------AISESTCEQELGLMSINEN--KCVGVPPEDQNVSGSDEVT-----DLPALNKSLVGASLMPESSSEPCILDQPAGPTNECLPKL-------YCPATKCPA : 199
MtCOLf   : FSGCPSASELSSIWPFFSDIP-----------------------------STGEA-CEHKLGLMSINENSDNSARVPPESKNVSGSAQVA-----DLPSKNKSGVDTSSIPESSAKPRILDQAPGSSNEFMPKL-------LCPSRKAPA : 200
MtCOLe   : YSGCPSSAELSSIWSFVLDIP-----------------------------SLSETTCEQELGFMSINEN--RSAWVDPKNQNVSDSDKAT-----DLPDLDKSFAGTSSMPESSKEPRMLDRPDGSTNECVPKL-------YCPATNCRE : 198
AtCOL11  : YSDCPSPSDFGKIWSSTLEP-------------------------------SVTSLVSPFSDTL--------------LQELDD-WNGSS-------------------TSVVTQ----TQNLKDYSSFFPMES-------N--LPKVIE : 162
AtCOL12  : FSGCPSPTDFNRMWSSILEP-------------------------------PVSGLLSPFVGSFP-------------LNDLNNTMFDTA-------------------YSMVPHNISYTQNFSDNLSFFSTES-------KGYPDMVLK : 170
GmCOL10a : YTGCPSLAEFSRIWSFVFDAN-----------------------------SSLGGWES--VGTLPKSESCTS--QCLEQTDHNGGSFGLS--------------------QIIPSNPNYTPFCKDEAFFFPQDS-------NQPKECPNL : 180
GmCOL10b : YTGCPSLAEFSRLWSFVFDAN-----------------------------SSLGGWES--VGTLPKSESCTS--QCLEQTDHNGGYFGLVSDKLDEIESCVRYQPRMDQSQIIPSNPNYTPYCKDEAFFFPQDS-------NQPKECPNL : 200
MtCOLj   : YTGCPSLAELSKIWPHLVDAN-----------------------------SSNAAWESPSTSSLPKTESSSGRGQHLEQQPEKNGFVGLANDKLGEGDTCVKYEPWIENSPIIPSNSNCTQYYKDQPFLFNQDS-------NQQK---DL : 201
GmCOL11a : FTGCPSVSELLSIVGFSDL--------------------------------SKKSLLSSPQGSGADGIFGG-EIEGLSDLFVWDSPSFVTLDDLISSSP-----------------------------------------------SSHS : 171
GmCOL11b : FTGCPSVSELLSIVGFSDI--------------------------------SKKSLLFSPQGSVADGFFGASEIEGLSDMFVWDAPSFVTLDDLISSSP-----------------------------------------------SSHS : 167
MtCOLg   : FTGCPSVTELLSILGLQDI--------------------------------GKKSLLL-PQESVGDGFVGY-EIEGLSDMFVWDAPSFVSLDDLISSSP-----------------------------------------------SSHN : 175
GmCOL12a : FSGCPSVTELLTILGLS-----------------------------------EKSLLSNEGTSQID--------DDLSDLHVWSAPSINGLK---------------------------------------------------------- : 148
GmCOL12b : FSGCPSVTELLTILGLS-----------------------------------EKSLLSNEGTSQID--------YDLSDLHESLASIFSGVNNFF------------------------------------------------------- : 153
AtCOL13  : FTGCPSVPELLAIVGLDDL--------------------------------TLDSGLLWESPEIVS----------LNDLIVSGGSGTHNFR---------------------------------------------------------- : 148
AtCOL14  : FSGCPSVLELAAVWGIDLK-GKKKEDDED----ELTKNFGMGLDSWGSGSNIVQELIVPYDVSCKKQS---FSFGRSKQVVFEQL-ELLKRGFVEGEGE------IMVPEGINGGGS-------ISQPSPTTS--------FTSLLMSQS : 219
AtCOL15  : FSGCPSALELAALWGLDLEQGRKDEENQVPMMAMMMDNFGMQLDSWVLGS---NELIVPSDTTFKKRGSCGSSCGRYKQVLCKQLEELLKSGVVGGDGDDGDRDRDCDREGACDGDGDGEAGEGLMVPEMSER--------LKWSRDVEE : 235
GmCOL13a : LSGCPSVTEIVSALCLDFR---------------------------------AQDPVVPTAASG------------GRDEVYEQVLEIAR-QRNDDLGA--------EQLKFDESPINDVVVVDEMLMQQTP--------------FTND : 172
GmCOL13b : LSGCPSVPEIASALGLDFR---------------------------------AQEPVVPTAAS--------------RDEVYEQVLEIAR-QRNNGLGA--------EQLKFDDSPGNDTPFTSLLMLQNSES--------EFDARKSND : 180
MtCOLh   : LTGCPSVHEIVSTLGLDLKPNDAVF------------------------VAEFEGPVVPVVKN--------------RDEVYEQVVEVAKRKRNLEEDQ--------NELRFNDCCND---VDDLLLLQQTPFTSLLNFSSEFDVGVKRN : 199
AtCOL6   : -----------------------------------------------SHTMVFHDLVPEMSTEDQAES-------YEVEEQLIFEVPVMNSMVEEQCFNQSLEKQNEFPMMPLSFKSSD-----EEDDDNAES----------------- :  98
AtCOL16  : -----------------------------------------------NNSSIFHDLVPDISIEDQTDN-------YELEEQLICQVPVLDPLVSEQFLNDVVEPKIEFPMIRSGLMIEE-----EED--NAES----------------- : 173
GmCOL7a  : ----------------------------------------------RNNN--PFHLVPEEGSEEANSH-------DENEEQLLYRVPIFDPFVAELCGTNSSPSPVTSTD--QGVVAAA-----EVEYKGFQS-----------NGFCS- : 174
GmCOL7b  : ----------------------------------------------RNNN--PFHLVPEEGSEEANSH-------DENEEQLVYRVPIVDPFVAELCGTNSSPSSVTSTD--QGVVAAAAAASAEVEYKGIQS-----------NDFCS- : 174
MtCOLk   : SKSSSSSEPA------------------------------------RNNNNIPFHLVPELGFDEVNSNSI-----EENEAQLLYRVPIFDPSIADLC---TSPPSVCSTEGGLGVVVVASAFAPDVKNNESESR----------VQLGSD : 193
GmCOL6a  : -SKHFALQQR-------LKDEVL-----------------------FNN-TSVLPLVPELGGEEQE--PVVVDNDET-EEQMLCRVPVFD--PFDVRTD---------------------------DLDSFS------------------ : 155
GmCOL6b  : -SKHFALQQR-------LKHEVL-----------------------FNN-TSVLPLVPELGGEEQE--PVVVDNDET-EEQMLCRVPVFD--PFDVRTD---------------------------DLDSFS------------------ : 158
MtCOLi   : NSSIQQQQQR-------LKEKVL-----------------------FN--TSFLPLVPELGGEEEQGQELLVDIDEADEEQLLCRVPVFDANPFDLETCTVKNDAVDFEE--------------MCDLDSFCE----------------- : 180
GmCOL5a  : HSSLKQQQQKKPLHEERGEEEVF-----------------------FNNTISLLPLVPELGSEEPL------LNDET-EEQLLCRVPVFDAELCSIYNEVKDEVVAAGEE--------------ALDLENFSS----------------- : 184
GmCOL5b  : HSSLQQQQQK-PLHEEQGEEE-------------------------------------ELGSEEPL------LNDET-EEQLLCRVPVFDAELS-------------------------------FDLENFSS----------------- : 151
AtCOL7   : FEKLLQIESN-------------------------------------------DPLVPELGGDEDDGFFS-FSSVEETEESLNCCVPVFDPFSDMLIDDINGFCLVPDEVNNTTTNGELG----EVEKAIMDD----------------- : 113
AtCOL8   : KPQQKIDDER----------------------------------------RREDPRVPEIGGEVMFFIP------EANDDDMTSLVPEFEGFTEMGFFLSN-------------------------HNGTEET----------------- : 168
```

```
                     *       320         *       340         *       360         *       380         *       400         *       420         *       440         *
AtCO      : NNNQNNGLLFS---DEYLN-LVDYNSSM--DYKFTG-------EYS---QHQQNCSVPQTSYGGDRVVPLKLEESRGH--------------------------------------------QCHN------QQNFQFNIKYG-SSGTHY : 245
AtCOL2    : IGNQNNGFLFG---VEYLD-LVDYSSSM--DNQFEDN------QYT---HYQR-------SFGGDGVVPLQVEESTSH--------------------------------------------LQQS------QQNFQLGINYGFSSGAHY : 217
AtCOL1    : SGN-NNGFSIG---DEFLN-LVDYSSS---DKQFTD-------QSN---QYQLDCNVPQRSYGEDGVVPLQIEVSKG---------------------------------------------MYQE------QQNFQLSINCG-SWGALR : 230
GmCOL1a   : NNNTNNGFSYNGEVDEYLD-LVDDC----DNHHFAS-VATTTDHYS--HQHQHFGVVSHKSYAGDSVVPVQHH------------------------------------------------------------QHFQLGLEFD-NSKAAF : 220
GmCOL1b   : NNNNNNGFLYNGEVDEYLD-LVDNCNSCGDNNHFAS-AAATTDHY---AQHQHFAGVSQKSYAGDSVVPVQQH------------------------------------------------------------QHFQLGLEFD-NSKPAF : 224
MtCOLa    : NQVANNGYLFSGEVDEYLD-LVDCNSWGGDENTFTTNNTHHHDEYSQQQQQQDHYGVPQKSYVGDSVVPVQQQ-----------------------------------------------------Q-----VQNFQLGLEFE-SSKAGF : 272
GmCOL2a   : N-GGDKGFLFG---DEYFDNLVD-CNSCGHNNNQFS------NVYD--QHQQNYSNTVPQNYA---VVPVQVP------------------------------------------------------------QHFQPGLDFD-SSKAGF : 235
GmCOL2b   : NDCGDEGFLFV---DEYLDNLVDCCNSCGHNDNQFS------NVY---QHQQNY-NTVPQNYV---VVPVQVP------------------------------------------------------------QHFQPGLDFD-SSKAGF : 240
GmCOL3a   : LNSS--------------QYLFSETEPVPYIDLDYA--------------AVDPKAEQKSSATADGVVPVQSNFEP--------------------------------------------------------FAYGYKYN--------TT : 199
GmCOL3b   : LNSS--------------QYLFSETEPVPYIDLDYA--------------AMDPKTEQKSSATADGVVPVQSNFEP--------------------------------------------------------FTYGYKYN--------TT : 199
MtCOLc    : LNSS--------------PYLFSDSEAIPFMDLDYG--------------VIEHKN--------DGVVPVHGNFDP--------------------------------------------------------FVSAYKNNNVHLHTELET : 191
MtCOLb    : LNYS--------------HYSYPEIDDFVTVNTKTD--------------LPEQNS---PGTTADGVVPVQSHSKT--------------------------------------------------------ATEHEHEHYSDINIDFSN : 198
AtCOL3    : ITN-----------------LFS------DLDYPKI--------------EVTSE---ENSSGNDGVVPVQNKLF---------------------------------------------------------LNEDYFNFDLS-ASK--I : 182
AtCOL4    : VTSAEEVPGDSPEMNTGQQYLFSDPDPYLDLDYGNV--------------DPKVESLEQNSSGTDGVVPVENRTVRIP-----------------------------------------------------TVNENCFEMDFTGGSKGFT : 281
GmCOL4a   : MDAPEIK---------SKEIFFSEMDPFLDFDYSNS--------------FQNHNS-----AVNDSVVPVQTKPSLAP--------------------------------------------PPINNHQHHHQSETCFDIDFCR-SKLSS : 240
GmCOL4b   : MDAPEIK---------SKEIFFSEMDPFLDFDYSNS--------------FQNNNS-----AGNDSVVPVQ-KPSLAP--------------------------------------------PLINNH-HHHQSETCFDVDFCR-SKLSS : 237
MtCOLd    : NETQDIK---------TREMFFSDMDPFLDFDYSNN--------------FQNNNCS---NAMNDSVVPVQTKPTPAP--------------------------------------------------MMNHNSEGCFDIDFCR-SKLSS : 235
AtCOL5    : IGTENMKG--------SSDFMFSDFDRLIDFEFPNS--------------FNHHQN----NAGGDSLVPVQTKTEPLP----------------------------------------------------LTNNDHCFDIDFCR-SKLSA : 233
AtCOL9    : MCEDDF--YGNLGMDEVDMALENYEELFG-TAFNPSEELFGHGGIDSLFH-------------KHQTAPEGG----------------------------------------------------------------NSVQPAGSN----D : 245
AtCOL10   : VCEDDF--NGNLISDEVDLALENYEELFG-SAFNSSRYLFEHGGIGSLFE-------------KDE-AHEG-----------------------------------------------------------------SMQQPALSNNASAD : 245
GmCOL8a   : ICEDDD-LYDDFIMDEVDLELENYEELFG-MALSHSEELFENGGINSLFEAKDMSASAGDSHCQGAVAAEGSSAGL-----------------------------------------------------------VNPIQPACSNAASAD : 283
GmCOL8b   : IYEDDD-LYDDFIMDEVDLQLENYEELFG-MALSHSEELFENGGINSLFETKDMSASAGDSHCQGAVAAEGSSAEL-----------------------------------------------------------VNAIQPACSNAASAD : 289
GmCOL9a   : LCENDN-LYDDFNMDEVDLNLENYEELFG-MALSHSEELFENGGIDSLFGTKDMSA--GDFSCEDAIAAEGSSVGQ-----------------------------------------------------------VNVMQPACSNAASAD : 286
GmCOL9b   : LSEDDN-LYDDFNMDEVDLDLENYEELFG-MALSHSEELFENGGIDSLFGTKGMSA--GDSNCQEAIAAEGSSVGQ-----------------------------------------------------------VNAMQPACSNAASAD : 286
MtCOLf    : LCEDDK-LLDDFNIDEMDFELENYSELFD-FALNHSEEFFENGGINSLFERKDMSASAGDSNCQGAFAAEGSSARF-----------------------------------------------------------VSAIQPECSNAASAD : 289
MtCOLe    : ASDDDDDLYGDFDMDEMDINMENYDELFG-MALTHSEELFENGGFNSLFGAKAMSA--GDSNCQDANAAEGSSIGH-----------------------------------------------------------VNAAQPACSTAASAD : 286
AtCOL11   : EECSGLDLCEGINLDDAPLNFNASNDIIGCSSLDNTK---------CYEYED----SFKE---ENNIGLPS------------------------------------------------------------------LLLPTLSG---NV : 227
AtCOL12   : LEEGEEDLCEGLNLDDAPLNFDVGDDIIGCSSEVHIEPDHTVP--NCLLIDK----TNTSSFTGSNFTVDK------------------------------------------------------------------ALEASPPGQQMNI : 248
GmCOL10a  : GIHDGSELCEGFNVDNLQLNFESADEIFG-CSQDATRYHLEDGGMDCLLMDKNISVTESNSLIESALEASSSIQQD-----------------------------------------------------------CVAFQSSRAGGSASV : 270
GmCOL10b  : GIHDGNELCEGFNVDNVQLNFEIADEIFY-CSQVATRYHLEDGGMDCLLMDKNIAVTESSSLIESAMEASSSIQQD-----------------------------------------------------------CVAFQSSRAGGSASV : 290
MtCOLj    : IIHEGTSLCEGFNVDDIQLNFESADEIIFDCSQTATKYNHEDGGIECLLMDKNIPVTKCSSHIETAVEASSSVQQD-----------------------------------------------------------CMIFPSSGAGGSTNL : 292
GmCOL11a  : FQAMEVPPLPKNRKAACGQHKEEILSQLRELAKSEP------LDLEPYVSSGNLSSGFEREPEADIFPSHEWHRES-----------------------------------------------------------SEPMYQVVPPDPLMR : 256
GmCOL11b  : FQAMKVPPLPKNRKAACGQHKEEILSQLRELAKSEP------LDLEPYVQSGNLSLGFERDPEADIFPSHEWHRES-----------------------------------------------------------SEPMHQVVPPDPSMG : 252
MtCOLg    : YRAMEVPPLPKNRKAACGRHREEILNQLREMTKSEP------YDPEEYIPPANLSTSFDCDVKADIVPSNEWLRES-----------------------------------------------------------SEPMYQVVPVDTSFK : 260
GmCOL12a  : ------------RKGACGRHKEEILSQLRELIKLEPDLIH----AERQGQFGNLPTDFERDVEANIFPSYECTNGS-----------------------------------------------------------LKRIIQIDERTIVAY : 223
GmCOL12b  : --IWLILLLNQNRKSAFGRHKEEILSQLRELIKLEPDLIHGEVDAERQGQFGNLPTGFERDVEASMFPSYE------------------------------------------------------------------------------- : 222
AtCOL13   : --ATDVPPLPKNRHATCGKYKDEMIRQLRGLSRSEPG------CLKFETPDAEIDAGFQF-LAPDLFSTCELESG------------------------------------------------------------LKWFDQQDHEDFPYC : 229
AtCOL14   : LCGNG-----MQWNAT--NHSTGQNTQIWDFNLGQSRNPDEPSPVETK------GSTFTFNNVTHLKNDTRTTNMN-----------------------------------------------------------AFKESYQQEDSVHST : 297
AtCOL15   : INGGGGGGVNQQWNATTTNPSGGQSSQIWDFNLGQSRGPEDTSRVEAAYVGKGAASSFTINNFVDHMNETCSTNVK-----------------------------------------------------------GVKEIKK-DDYKRST : 325
GmCOL13a  : NNGYGTEAGDLLWNYN----PAYQPPQVWDFQLQKSRDCHE-PRVVTF---DGLEVPKLFQDEHNMKYSTIGDDID-----------------------------------------------------------ILSRNNQSDQSSSSH : 255
GmCOL13b  : NNGYGTEAWDLHWNYNN---PAYQPPQVWDFQLQKSTDCNE-PRV----------------DVHNMNYSTIGDDID-----------------------------------------------------------ILSRNNQSDQSSSSH : 251
MtCOLh    : SNDYGNESGLLLWDRN----PSYQPPQVWDFQLQKSRDMTY-DGVEN----ASLSIPKSLQDVHNMNCSTLGD--D-----------------------------------------------------------ILSRNNQSDQSSSSH : 279
AtCOL6    : ------CLNGLFPTDMELAQFTADVETLLG-GGDREFHSIEELGLG--EMLKIEK----EEVEEEGVVTREVHDQD----EGDETSPFEISFDYEYTHKTTFDEGEEDEKEDVMKNVMEMGVNEMSGGIKEEKKEKALMLRLDYESVIST : 231
AtCOL16   : ------CLNGFFPTDMELEEFAADVETLLGRGLDTESYAMEELGLSNSEMFKIEKDEIEEEVEEIKAMSMDIFDDDR--KDVDGTVPFELSFDYESSHKTS--------EEEVMKNVESSGECVVK--VKEEEHKNVLMLRLNYDSVIST : 305
GmCOL7a   : NSSEMENLHGMLPSDAELAEFAADVESLLGRGLENECVGMEELGLVDAKEEECS--VGSGKVKMEDQEESPLVEMEMDMVVGRDDQSFELSFDYETCEEV------KVCDLGLGNELGAKKENDD------EVKKNKISLQLDYEAVIIA : 310
GmCOL7b   : NSNEIENLHGMLPSDAELAEFAADVESFLGRGLENKCVGMEELGLVDTKEEECS--VGSGKVKVEE-EESPLMEMDM----GRDDQSFELSFDYETCEEVK---EMKVSDLELGNELGEMKENDDD-----EVKRKKVSLQLDYEAIIIA : 309
MtCOLk    : NNYEMESFHGLLPSDIELAEFAADVESLLGRGLENECIGMEELGLIDTKHEESEKWKCGGKVKEEGEECYEVVEGDNMMEIGKES-SFELNFDYDDSHET---------CEEVKEKCGEQNENNDY-----NKGKRKISLQLDYDAVIIA : 328
GmCOL6a   : --------------DMDFAEFAADVESLLDKEDDE--ISACVGG---GEGVQGAMVKVKDEE-EIDGDVACYLESVF-------DDAFHWNNIESVLSDAR--------EEKEGVVACDGGVGD-----EEGGTKRDIFLRLNYDEVITA : 265
GmCOL6b   : --------------DMDFSEFAADVEGRLDKEDDE--ISAYVGG---GEGVQGVLAKVKGEE-EIDGDVACYLESVFD---MISDDAFHWNNIESVVSDAR--------EEKGCVVPCDGGVS------EQGGIKRDIFLRLNYDEVITA : 271
MtCOLi    : ---------F----DVDLAEFAANVESLLGVGSSE--IQENSSGQVFDYKQENEMDASKSEMLKVKDEELDDLESVFD---MTSDDVFHWNIDNNDVSLAQ--------QEKEYMPLSNSSVGYSESVITKEETKRERFLRLNYEEVITE : 304
GmCOL5a   : ---------EFLPSDTDLAEFTADVKSFLGNGADE--DSPDDDE----HVKESELLILDCKEEEGDEEMDECIDGVL------------IGA-KDAMVK-------------------------------------DIFLRLNYEEVITA : 269
GmCOL5b   : ---------EFLPTDMDLAEFSADVESLLGNGVDE--DSPVK--------KGSELVLLDCKEE-GDDEMDACVNG--------------IGANKDAMVKVK--------DEEELDADDDTACHL------------DSILDMNSEAFNWN : 247
AtCOL7    : -----EGFMGFVPLDMDLEDLTMDVESLLEEEQLCLGFKEPNDVGVIKEENKVGFEINCKDLKRVKDEDEEEEEAKCE-------NGGSKDSDREASNDKD--------------------------------RKTSLFLRLDYGAVISA : 219
AtCOL8    : ------------TKQFNFEEEADTMEDLYYNGEEEDKTDGAEACPGQYLMSCKKDYDNVITVSEKTEEIEDCYENNAR-------------------------------------------------------------HRLNYENVIAA : 245
```

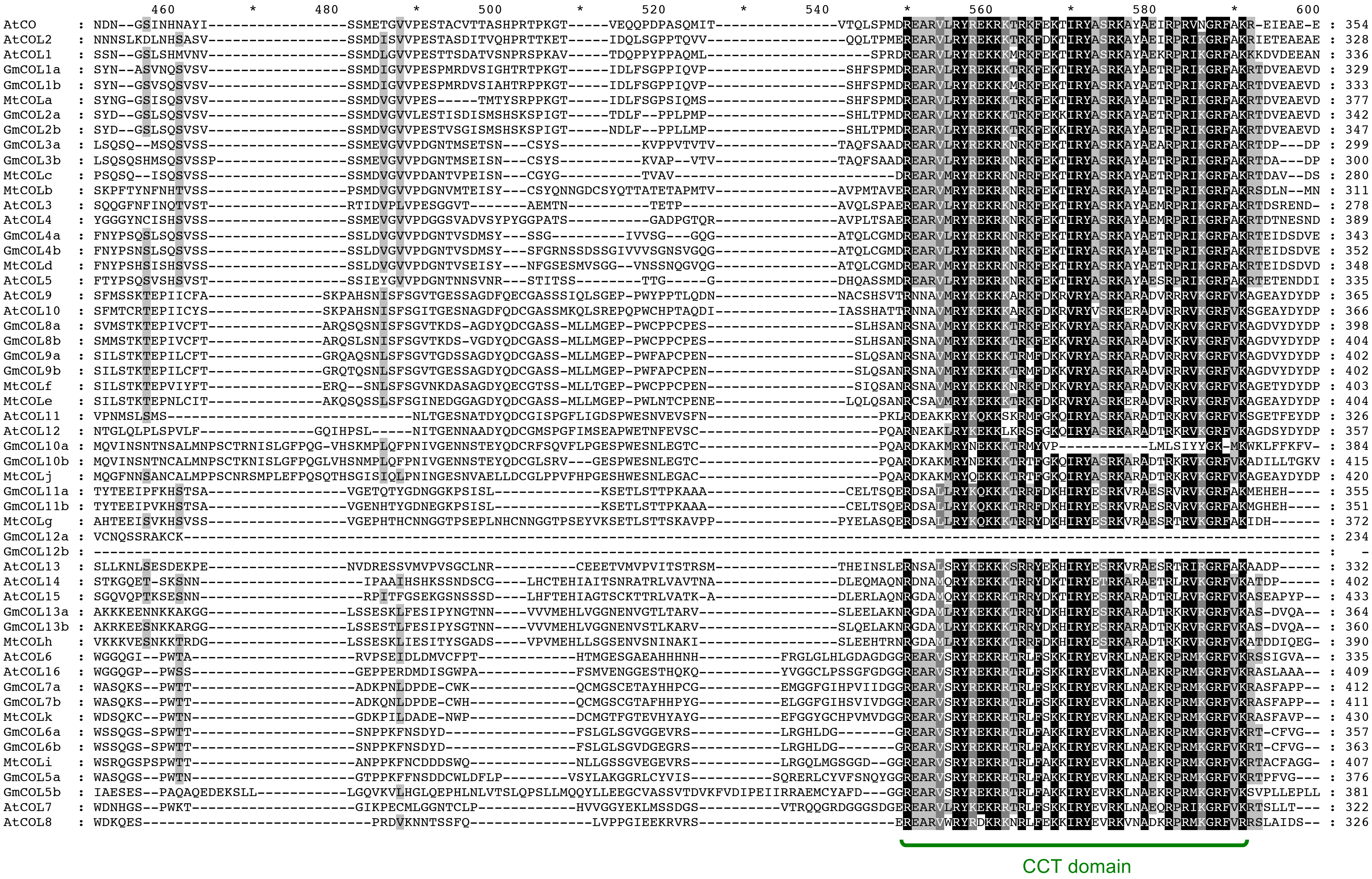
CCT domain

```
                    *       620         *       640
AtCO     : QGFNTM--------LMYNTGYGIVPSF---------------------- : 373
AtCOL2   : EIFSTS--------LMSETGYGIVPSF---------------------- : 347
AtCOL1   : QAFSTM--------ITFDTGYGIVPSF---------------------- : 355
GmCOL1a  : QMFSTT--------LITEVGYGIVPSF---------------------- : 348
GmCOL1b  : QMFSTT--------LITEVGYGIVPSF---------------------- : 352
MtCOLa   : QMFSTS--------LITEVGYGIVPSF---------------------- : 396
GmCOL2a  : QMFSTT--------LFTEVGGSIFPTF---------------------- : 361
GmCOL2b  : QMFSTK--------LFNEVGGSIFPTF---------------------- : 366
GmCOL3a  : LAG-----------------YGVVPSC---------------------- : 309
GmCOL3b  : LAG-----------------YGVVPSC---------------------- : 310
MtCOLc   : ISG-----------------YGVVPTC---------------------- : 290
MtCOLb   : LIAED--------------EYGVVPSC---------------------- : 324
AtCOL3   : -GGDVG----------VYGGFGVVPSF---------------------- : 294
AtCOL4   : VVGHGG----------IFSGFGLVPTF---------------------- : 406
GmCOL4a  : RLYSPG-----AAALMLDTPYGVVPTF---------------------- : 365
GmCOL4b  : RLYSPG-----PAVLMLDTPYGVVPSF---------------------- : 374
MtCOLd   : RLYNPADPLSVPSSMLMDCPYGVVPTF---------------------- : 375
AtCOL5   : FLSHVY-------ASAAHAQYGVVPTF---------------------- : 355
AtCOL9   : LTPTRSY------------------------------------------ : 372
AtCOL10  : MSPTRSY------------------------------------------ : 373
GmCOL8a  : LNQTRSY------------------------------------------ : 405
GmCOL8b  : LNQTRSC------------------------------------------ : 411
GmCOL9a  : LSTTRSF------------------------------------------ : 409
GmCOL9b  : LSTTRSC------------------------------------------ : 409
MtCOLf   : LSQTRSC------------------------------------------ : 410
MtCOLe   : LSQTRSY------------------------------------------ : 411
AtCOL11  : SLVM--------------------------------------------- : 330
AtCOL12  : SSPTTNN------------------------------------------ : 364
GmCOL10a : ------------------------------------------------- :   -
GmCOL10b : VPGPCEYIALLPILHQQTFCLSCESGRVIMQLDIYSKLDCLGLDIPPCI : 464
MtCOLj   : LLSDH-------------------------------------------- : 425
GmCOL11a : ------------------------------------------------- :   -
GmCOL11b : ------------------------------------------------- :   -
MtCOLg   : ------------------------------------------------- :   -
GmCOL12a : ------------------------------------------------- :   -
GmCOL12b : ------------------------------------------------- :   -
AtCOL13  : ------------------------------------------------- :   -
AtCOL14  : ------------------------------------------------- :   -
AtCOL15  : ------------------------------------------------- :   -
GmCOL13a : ------------------------------------------------- :   -
GmCOL13b : ------------------------------------------------- :   -
MtCOLh   : ------------------------------------------------- :   -
AtCOL6   : ------H------------------------------------------ : 336
AtCOL16  : ------ASPLGVNY----------------------------------- : 417
GmCOL7a  : ------TFPLLNK------------------------------------ : 419
GmCOL7b  : ------TFPLLNK------------------------------------ : 418
MtCOLk   : ------TFPLLK------------------------------------- : 436
GmCOL6a  : ------ANAFPA-YH---------------------------------- : 365
GmCOL6b  : ------ANAFPA-YH---------------------------------- : 371
MtCOLi   : ------ATSFPTNYH---------------------------------- : 416
GmCOL5a  : ------ATALPA------------------------------------- : 382
GmCOL5b  : CQLNYQSSPLT-------------------------------------- : 392
AtCOL7   : ------------------------------------------------- :   -
AtCOL8   : ------------------------------------------------- :   -
```

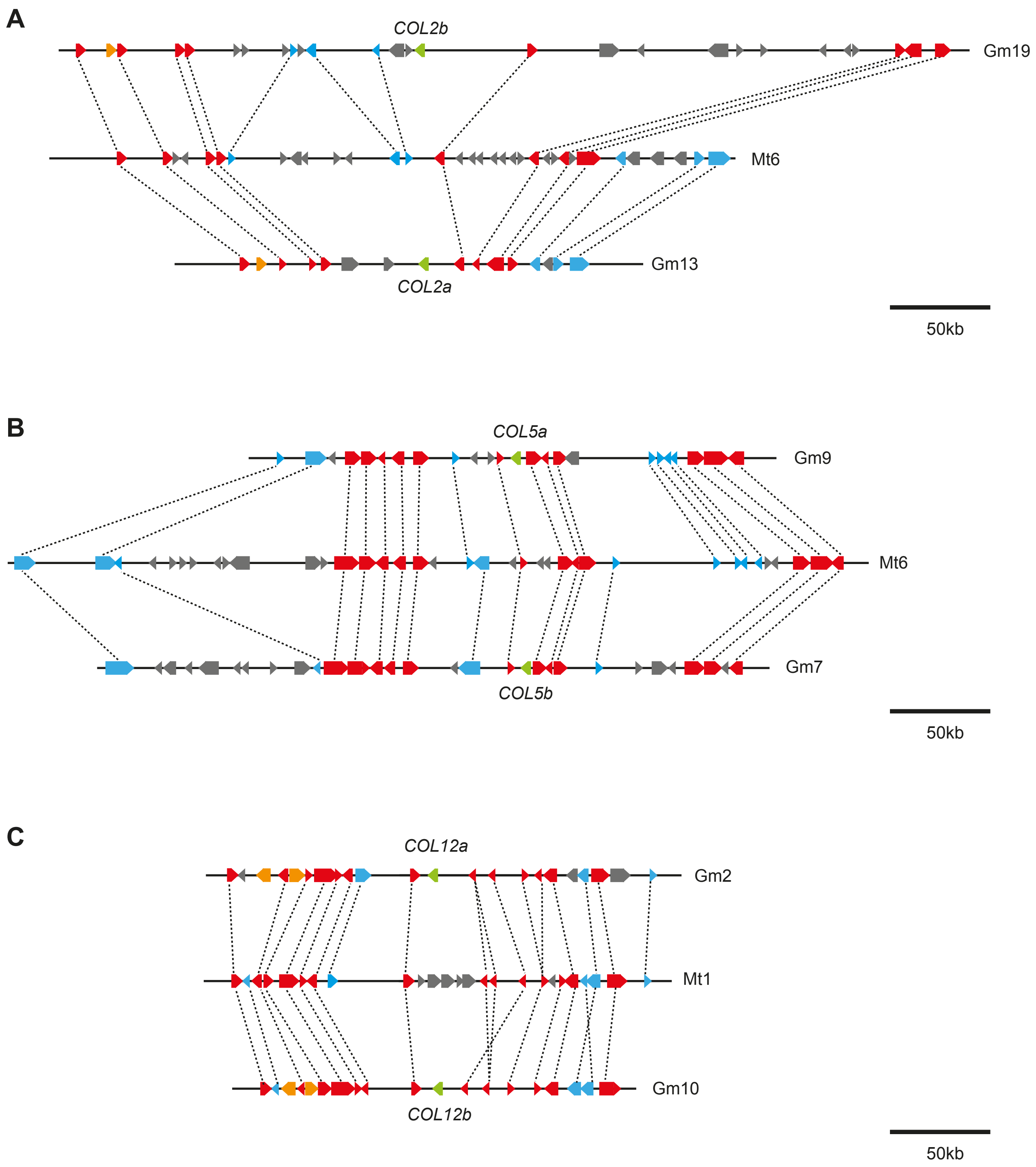

Supplemental Figure 2. Microsynteny between soybean and Medicago in the regions of soybean genes COL2a/b (**A**), COL5a/b (**B**) and COL12a/b (**C**), based on annotated genes in Mt v4.0 and Gm v2.0. Genes sharing the same identity (as determined by top ranking in reciprocal BLASTp searches in Phytozome) are connected by dashed lines. Soybean COL genes are shown in green. Genes shown in red are common to Medicago and both soybean homeologs, blue indicates genes common only to Medicago and one soybean homeolog, orange indicates genes common to both soybean homeologs but not present in Medicago, and all other genes with no obvious match within the depicted region are shown in grey.

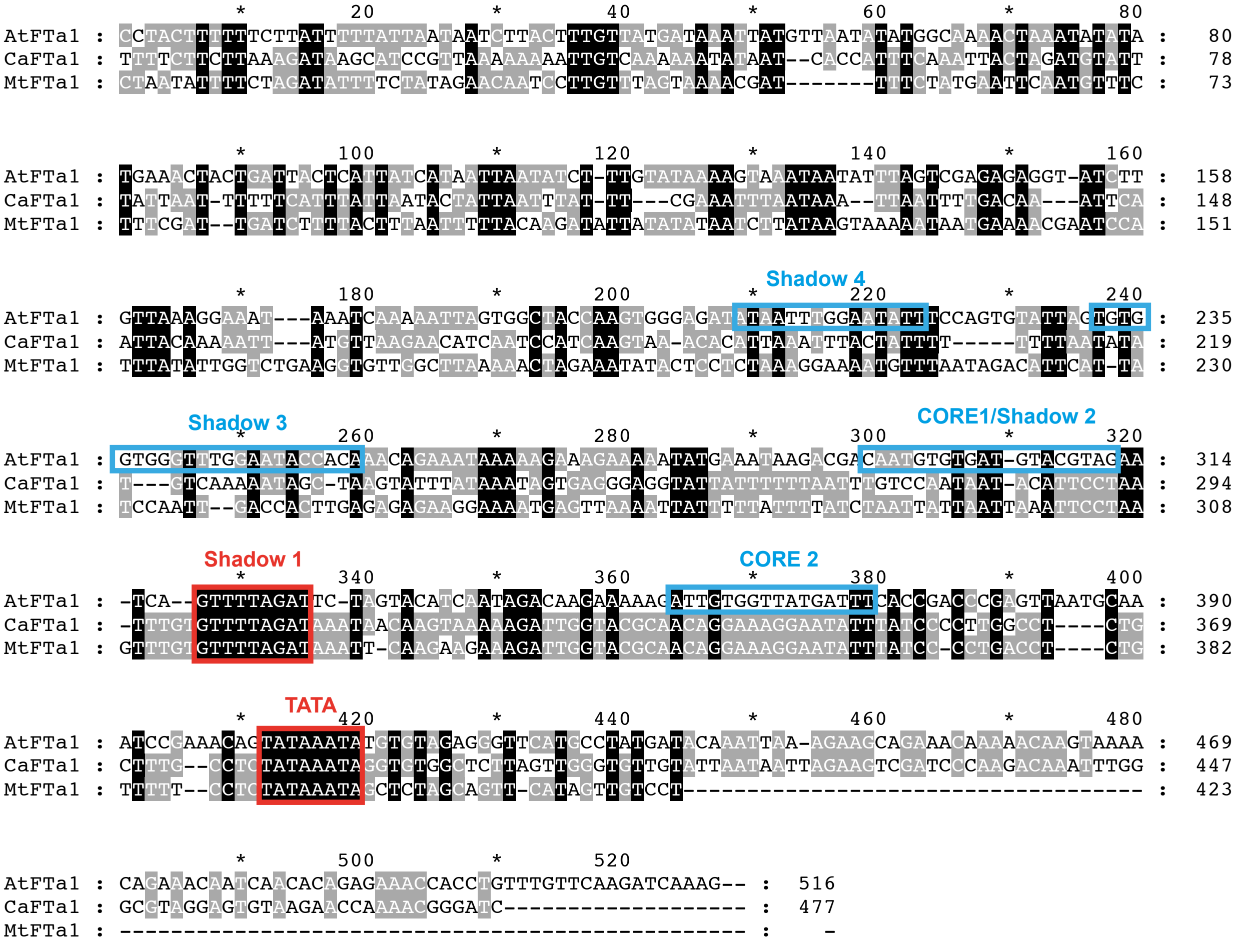

Supplemental Figure 3. Alignment of the proximal promoters of the Arabidopsis *CO* gene and the *FTa1* genes from Medicago and chickpea. Conserved or functionally significant regions in the Arabidopsis *FT* promoter identified in previous studies (Adrian et al., 2010; Tiwari et al., 2010) are indicated. Only the Shadow1 and putative TATA boxes show significant conservation, whereas the CO-responsive elements CORE1 and CORE2 and other shadow regions do not appear significantly conserved.

Supplemental Table 1. Identities of *COL* genes included in Figure 1 and Supplemental Figure 1.

	**Soybean**			**Medicago**	
	Name (Wu)	Name (Fan)	Gm1.1 gene model	Name	Mt4.0 gene model
Group Ia	GmCOL1a	GmCOL1	Glyma08g28370	MtCOLa	Medtr7g018170
	GmCOL1b	GmCOL2	Glyma18g51320		
	GmCOL2a	GmCOL5	Glyma13g07030	not present	
	GmCOL2b	GmCOL13	Glyma19g05170		
Group Ic	?	?	?	MtCOLb	Medtr1g013450
	?	?	?		
	GmCOL3a	GmCOL14	Glyma04g06240	MtCOLc	Medtr3g105710
	GmCOL3b	GmCOL3	Glyma06g06300		
	GmCOL4a	GmCOL10	Glyma13g01290	MtCOLd	Medtr4g128930
	GmCOL4b	GmCOL8	Glyma17g07420		
Group II	GmCOL5a	GmCOL21	Glyma07g10160	not present	
	GmCOL5b	GmCOL23	Glyma13g33420		
	GmCOL6a		Glyma05g35151	MtCOLi	Medtr8g104190
	GmCOL6b	GmCOL19	Glyma08g04570		
	GmCOL7a	GmCOL22	Glyma10g42090	MtCOLk	Medtr1g110870
	GmCOL7b	GmCOL6	Glyma20g24940		
Group III	GmCOL8a	GmCOL18	Glyma02g38870	MtCOLe<br>MtCOLf	Medtr3g082630<br>Medtr5g069480
	GmCOL8b	GmCOL7	Glyma14g36930		
	GmCOL9a		Glyma13g15592		
	GmCOL9b		Glyma20g07051		
	GmCOL10a	GmCOL12	Glyma12g32220	MtCOLj	Medtr2g088900
	GmCOL10b	GmCOL20	Glyma13g38250		
	GmCOL11a	GmCOL15	Glyma03g36810	MtCOLg	Medtr7g108150
	GmCOL11b	GmCOL9	Glyma19g39460		
	GmCOL12a	GmCOL28	Glyma02g17180	not present	
	GmCOL12b	GmCOL27	Glyma10g02620		
	GmCOL13a	GmCOL16	Glyma16g05540	MtCOLh	Medtr7g083540
	GmCOL13b	GmCOL17	Glyma19g27240		

Supplemental Table 2. Primers used for genetic and expression analyses

Gene	Primer Name	Primer Sequence	Tm (ºC)
Tnt1	Tnt1-F	ACAGTGCTACCTCCTCTGGATG	60
	Tnt1-R	CAGTGAACGAGCAGAACCTGTG	60
TEF1	MtTEF1-RT-F	CTTATCATTGACTCCACCACTG	56
	MtTEF1-RT-R	ACTTCCTTCACGATTTCATCGTA	
COLa	MtCOLa-F	CTTCCTTAACACTGTCTGTC	60
	MtCOLa-R	GGTGATTATGTCAAACTCACCG	60
	MtCOLa-RT-F	CGAAGATGAAGGCTTCGTCC	62
	MtCOLa-RT-R	AGCCACTTGATTATGATCATTGC	
COLb	MtCOLb-RT-F	CGGTGCAGTCTCATAGCAA	58
	MtCOLb-RT-R	CATCCATCGACGGTGAAGA	
COLc	MtCOLc-RT-F	TCACGGTAACTTCGATCCTTT	55
	MtCOLc-RT-R	CCGTAACCACAGTTTGA	
COLd	MtCOLd-RT-F	GCCGATGATGAATAACAACTCAG	62
	MtCOLd-RT-R	GTGAAGATGACGAAACGCTATG	
COLe	MtCOLe-qF3	TTTGGATAAGTCTTTCGCCG	58
	MtCOLe-qR3	TTCACGGCAATTTGTAGCAG	
COLf	MtCOLf-qF2	CAGTGCTAGGGTTCCTCCAG	62
	MtCOLf-qR2	CTGGAGCCTTTCTACTGGGA	
COLg	MtCOLg-qF2	TGCTTTTGCCTCAGGAAAGT	60
	MtCOLg-qR2	TGTGTCTACCACAAGCAGCC	
COLh	MtCOLh-qF5	TCCGTCGTATCAACCTCCTC	60
	MtCOLh-qR3	CACCAAGTGTTGAGCAATTCA	
COLi	MtCOLi-qF1	AGGCAAGGTTCTCCTTCTCC	62
	MtCOLi-qR1	CCTCCATCTCCACCACTTCC	
